# Propionate production by *Bacteroidia* gut bacteria and its dependence on substrate concentrations differs among species

**DOI:** 10.1186/s13068-024-02539-9

**Published:** 2024-07-10

**Authors:** Carolin Döring, Mirko Basen

**Affiliations:** 1 Department of Microbiology, Institute of Biological Sciences, University of Rostock, Albert-Einstein-Straße 3, 18059 Rostock, Germany; 2 Department of Maritime Systems, Interdisciplinary Faculty, University of Rostock, Rostock, Germany

**Keywords:** Bacteroidetes, Bacteroidota, Bacteroidia, Gut bacteria, Propionic acid fermentation, Propionate, Bacteroides graminisolvens, Bacteroides propionicifaciens

## Abstract

**Background:**

Propionate is a food preservative and platform chemical, but no biological process competes with current petrochemical production routes yet. Although propionate production has been described for gut bacteria of the class *Bacteroidia*, which also carry great capacity for the degradation of plant polymers, knowledge on propionate yields and productivities across species is scarce. This study aims to compare propionate production from glucose within *Bacteroidia* and characterize good propionate producers among this group.

**Results:**

We collected published information on propionate producing *Bacteroidia,* and selected ten species to be further examined. These species were grown under defined conditions to compare their product formation. While propionate, acetate, succinate, lactate and formate were produced, the product ratios varied greatly among the species. The two species with highest propionate yield, *B. propionicifaciens* (0.39 g_pro_/g_gluc_) and *B. graminisolvens* (0.25 g_pro_/g_gluc_), were further examined. Product formation and growth behavior differed significantly during CO_2_-limited growth and in resting cells experiments, as only *B. graminisolvens* depended on external-added NaHCO_3_, while their genome sequences only revealed few differences in the major catabolic pathways. Carbon mass and electron balances in experiments with resting cells were closed under the assumption that the oxidative pentose pathway was utilized for glucose oxidation next to glycolysis in *B. graminisolvens*. Finally, during pH-controlled fed-batch cultivation *B. propionicifaciens* and *B. graminisolvens* grew up to cell densities (OD_600_) of 8.1 and 9.8, and produced 119 mM and 33 mM of propionate from 130 and 105 mM glucose, respectively. A significant production of other acids, particularly lactate (25 mM), was observed in *B. graminisolvens* only.

**Conclusions:**

We obtained the first broad overview and comparison of propionate production in *Bacteroidia* strains. A closer look at two species with comparably high propionate yields, showed significant differences in their physiology. Further studies may reveal the molecular basis for high propionate yields in *Bacteroidia*, paving the road towards their biotechnological application for conversion of biomass-derived sugars to propionate.

**Supplementary Information:**

The online version contains supplementary material available at 10.1186/s13068-024-02539-9.

## Background

**Fig. 1:**
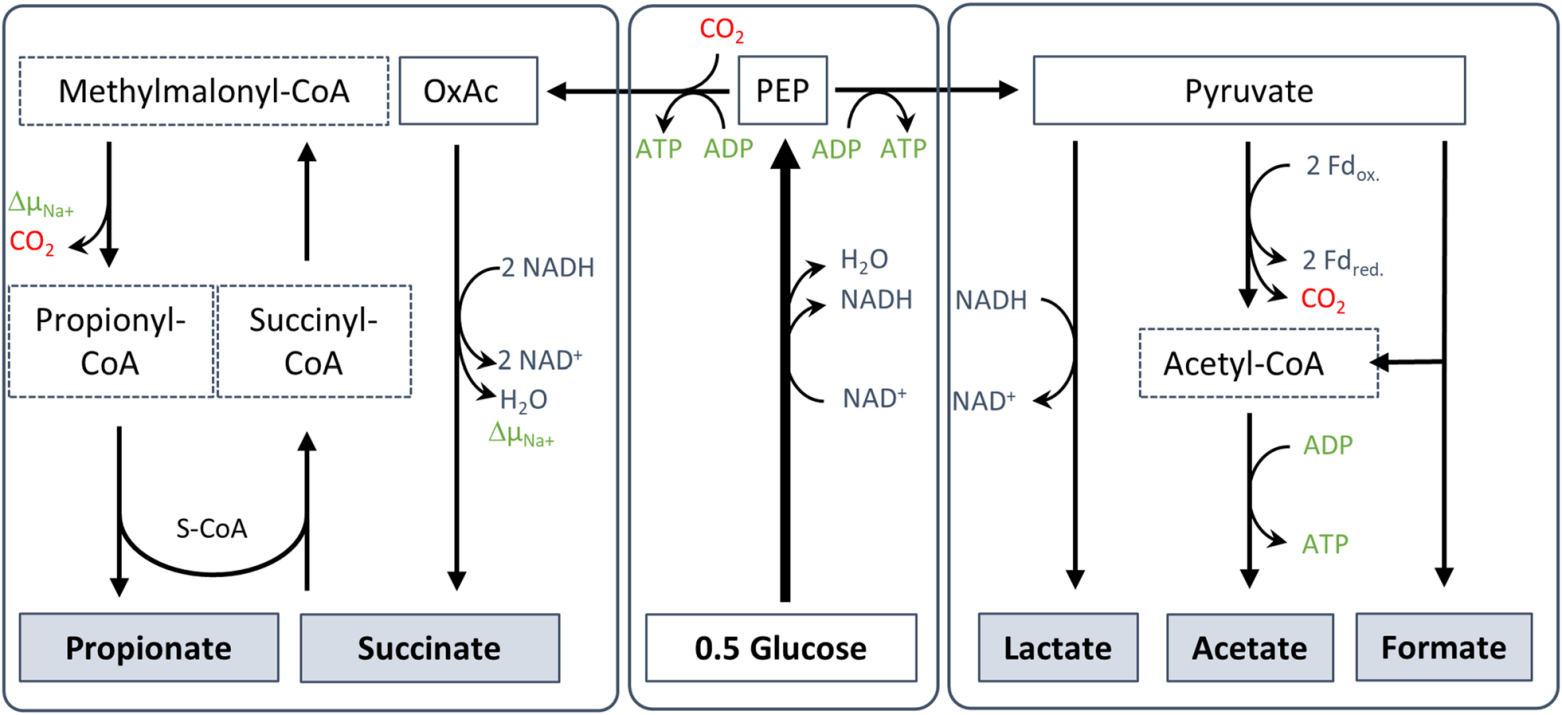
General overview of propionate production from glucose in *Bacteroidia*. Glucose is metabolized through the glycolysis into NADH and PEP. The latter can then either be carboxylated to oxaloacetate (OxAc) by PEP carboxykinase (PEP-CK) or converted into pyruvate. OxAc enters the reductive branch of the TCA and is reduced to succinate via fumarate and malate. Succinate can be converted further into propionate, which includes a decarboxylation. During conversion of oxaloacetate to propionate involving enzymes of the reductive TCA and the succinate pathway, a sodium gradient (Δµ_Na+_) is generated. Acetyl-CoA is either produced through the activity of the pyruvate:ferredoxin oxidoreductase (PFOR), which generates reduced ferredoxin and CO_2_, or through the pyruvate-formate lyase (PFL), which generates formate. Acetyl-CoA can then be converted into acetate, yielding ATP. Lactate is produced by reduction of pyruvate by lactate dehydrogenase (LDH) [19, 20, 23]

Propionic acid and its salts are valuable chemical compounds. Because of their antimicrobial and antifungal properties, they are widely utilized as preservative for animal feed as well as foods, such as bakery products [1, 2]. Moreover, propionate is gaining increasing attention as a platform chemical for the production of cellulose derived plastics, such as cellulose acetate propionate (CAP), cosmetics, pharmaceuticals and more [3]. While propionic acid is sold for about 1–2 € per kg, its derivates can reach much higher prices of up to 600 €/kg [4]. About 463,000 t were traded in 2022 worldwide and the market volume is forecasted to increase to around 600,000 t in 2030 [5]. The main part of the annual propionate production is derived from petroleum based feedstocks for example through the Reppe-process from ethylene, CO and steam or the Larson-process from ethanol and CO [6, 7]. However, these processes need fossil resources, which are highly dependent on fluctuating oil prizes. They also rely on expensive catalyst or high temperatures and pressure, and, therefore, a high energy input [3, 8]. In addition, consumers long for more naturally derived products from renewable resources. Production of propionate from renewable resources such as plant-based polymers would contribute to a sustainable and circular economy according to the Sustainable Development Goals of the UN [8]. As explained below, the current biological route from sugar to propionate is almost CO_2_ neutral (Fig. 1), and it is CO_2_-negative, considering CO_2_ fixation by the plant. Agricultural waste rather than corn cobs/starch may be used as resource to avoid competition for land between bioeconomy and food production. The use of microorganisms or their enzymes as bio-catalysts to convert plant-based and other biopolymers may also save costs, since they operate at lower temperatures and ambient pressures. Finally, using microorganisms as whole-cell biocatalysts, e.g., for the production of propionate from plant-based may be of more efficient and less costly than enzymatic biomass pretreatment [9].

Many studies were looking into biological propionate formation, which is widespread among bacteria. Fermentative pathways include the succinate pathway, the acrylate pathway or the 1,2-propanediol pathway [10], that can be found in genera, such as *Propionibacterium*, *Clostridia*, *Veillonella* and *Bacteroides*, but most studies regarding bacterial propionate formation focus on propionibacteria, such as *P. freudenreichii* or *A. acidipropionici*, which are believed to have the most efficient propionate production pathway and are intensively studied [6, 10]. Other strategies involve genetically engineered strains, that would otherwise not produce propionate, for example an *E. coli* strain carrying the sleeping beauty mutase, which converts succinate to propionate [11, 12], or a strain of *C. saccharoperbutylacetonicum*, using the acrylate pathway [13]. To compete with current production methods, a biological fermentation from glucose should meet certain benchmarks including a yield of 0.6 g_pro/_g_gluc_, a titer of 100 g/l and a productivity of 1—2 g/l*h [10]. Due to the formation of side products, as well as inhibitory effects of the product on the process itself, none of the efforts have met these fermentation goals simultaneously so far.

*Bacteroidia* have been intensively studied for their role in gut-health. In the bowel they can reduce inflammation reactions [14], but act as opportunistic pathogens in other body parts [15]. Some specific strains are even correlated to bowel inflammation and cancer development [16]. Although their role for host health is not always clear, it has long been recognized, that *Bacteroidia* fill a specific niche in the gut, as they are known to utilize a wide variety of polymers that are otherwise indigestible to the host, including fructans, pectins and hemicelluloses [17]. The polymers are hydrolyzed and, under anoxic conditions, fermented to a variety of acids such as propionate, succinate, acetate, lactate and formate, which in turn affect the health of the host. Especially propionate was found to have anti-inflammatory effects, increase Ca^2+^ uptake and increase satiety through activation of G-protein-coupled receptors [18].

The ability of *Bacteroidia* to utilize plant polymers, renders them interesting organisms for a possible biotechnological conversion of agricultural wastes to propionate, which would decrease production cost and, therefore, lower the benchmark for an economically feasible process. While studies have looked into the use of agricultural byproducts for propionate production through propionibacteria, substrates had to be pretreated to be accessible for those bacteria, for example, through enzymatic treatment, adding to production cost and thus increasing the benchmark for a competitive process [6].

*Bacteroidia* utilize the succinate pathway for propionate production (Fig. 1) [19]. In this, glucose is converted to pyruvate or phosphoenolpyruvate (PEP) and then to propionate via the intermediate succinate. Because of requirement of NADH for the reduction of oxaloacetate to succinate, acetate is produced from pyruvate oxidation by pyruvate:ferredoxin oxidoreductase (PFOR) as a side product. When all of the glucose is converted to acetate and propionate, a ratio of 2:1 can be expected, whereas formate and lactate production would decrease this ratio [10]. On top of their polymer degrading capacities, *Bacteroidia* are a compelling group to study for biological propionate production. First, most studies focusing on propionate production of *Bacteroidia* are outdated as many new species have been isolated in recent years and previous evaluation needs to be revised. In addition, recent genetic and physiological studies revealed details on metabolic pathways and bioenergetics of two representative species, *Segatella copri* and *Phocaeicola vulgatus* [20–23]. Finally, progress is made on the genetical accessibility of different *Bacteroidia* species [24–27], opening up opportunities to increase productivity, titer and yield in propionate fermentations.

Initially, we screened the literature for propionate producing *Bacteroidia* to identify strains with high propionate productivities and yields. However, we found that only few strains have been studied so far. Therefore, we selected a variety of strains known to produce propionate and systematically studied propionate production from glucose under defined conditions. The two most promising strains out of ten were selected for studies with resting cells and in bioreactors towards understanding the parameters and bottlenecks that control propionate production.

## Results and discussion

### Propionate production among Bacteroidia

Various novel *Bacteroidia* strains have been isolated and described over the last 20 years and propionate formation was found in many of them. To gain an overview of these species, a literature search was performed and information on propionate producing strains is collected in Table S1 (Additional file 1). Many of these strains utilize one or more polymeric substrates, such as xylan, starch or pectin, but organic acid production has only been described very briefly in most studies without stating product concentrations. Culture conditions among studies that did quantify propionate vary greatly, making it impossible to compare results. Finally, propionate production in *Bacteroides* has been described to be dependent on certain culture conditions, for example addition of vitamin B_12_ [28] and media have not always been optimized for propionate formation. Therefore, a first comparative screening was carried out on a simple sugar, glucose, to gain an overview of the product spectrum of an assortment of isolates. For this first screening, ten species were selected (Table S1, bold writing). Among them were 6 species of the genus *Bacteroides* as well as two species each of the genera *Parabacteroides* and *Phocaeicola*. Since increased laboratory safety measures increases costs for biotechnological processes, we solely focused on species that belong to risk group 1 according to the German technical rule for biological agencies 466 (TRBA). Unfortunately, a well-studied propionate producer, *Xylanibacter ruminicola* [29] (formerly *Prevotella ruminicola* [30]) belongs to risk group 2. The species were selected either because they utilized a wide spectrum of polymeric substrates, for example *B. cellulosilyticus*, which had been described to degrade crystalline cellulose [31], or because comparably high propionate concentrations were reported. While we aim to convert plant polysaccharides as renewable resource using *Bacteriodia* in the future, the current study focused on propionate production from glucose.

### Growth and propionate production of ten selected Bacteroidia strains

Although members of the class *Bacteroidia* have been known to produce propionate and other organic acids, no broad comparative studies that focus on propionate production have been performed. A growth experiment was performed to compare the selected species. The growth was examined with a defined minimal medium with glucose (DMMG) as well as DMMG-Y containing a low amount of yeast extract (0.5 g/l) to determine whether strains needed additional nutrients. The highest OD_600_ (OD_max_) was reached by *P. paurosaccharolyticus* and *P. vulgatus* with 0.92 ± 0.02 (DMMG-Y, Table S2, additional file 2). In general, OD_max_ was not affected by addition of yeast extract except for *B. propionicifaciens* and *P. johnsonii* for both of which OD_max_ increased by 0.08 in DMMG-Y compared to DMMG. Growth rates varied greatly among the species. The fastest growth occurred in *B. xylanisolvens*, which reached a growth rate of 0.50 h^−1^ without and 0.61 h^−1^ with the addition of yeast extract. The slowest growth rate was determined for *B. propionicifaciens*, which only reached a rate of 0.09 h^−1^ without and 0.14 h^−1^ with yeast extract. Because of its slow growth, the measurement had to be extended to 45 h to expand the growth curve to stationary growth phase. The growth rates of some species were not affected by yeast extract, namely *B. graminisolvens*, *B. luti*, *P. chartae,* and *P. paurosaccharolyticus*. This is interesting in the light of a potential biotechnological application, as the omission of complex compounds may reduce operation costs. An increase of the growth rates with addition of yeast extract was detected in all other strains, showing that there is variation in the nutrient need among this group of bacteria.

**Fig. 2:**
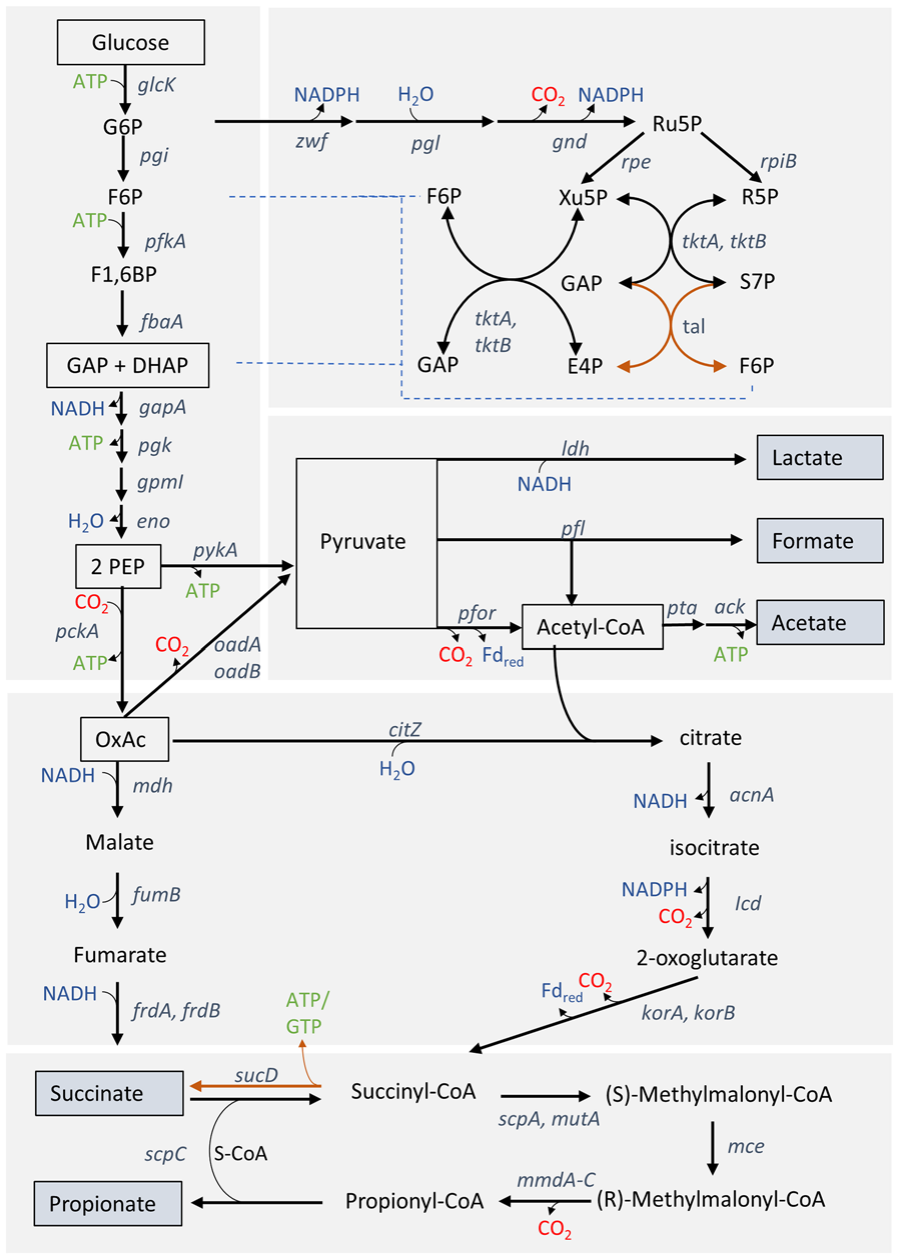
Proposed routes of the central carbon metabolism in the strains *B. graminisolvens* XDT-1 an *B. propionicifaciens* SV434. The presence of genes was determined by BLASTn analysis with two genomes each and amino acid sequences of characterized proteins. Genes that were only identified in *B. graminisolvens* are shown as orange arrows. Abbreviations: G6P (glucose 6-phosphate), F6P (fructose 6-phosphate), F1,6BP (fructose 1,6-bisphosphate), GAP (glyceraldehyde 3-phosphate), DHAP (dihydroxyacetone-phosphate), PEP (phosphoelolpyryvate), OxAc (oxaloacetate), Ru5P (ribulose 5-phosphate), Xu5P (xylulose 5-phosphate) R5P (ribose 5-phosphate), S7P (seduheptulose 7-phosphate), E4P (eythrose 4-phosphate). Genes: glucokinase (*glcK*), glucose-6-phosphate isomerase (*pgi*); 6-phosphofructokinase (*pfkA*), fructose 1,6-bisphosphate aldolase (*fbaA*), G6P dehydrogenase (*zwf*), 6-phosphogluconolactonase (*pgl*), phosphogluconate dehydrogenase (*gnd*), ribulose 5-phosphate 3-epimerase (*rpe*), ribose-5-phosphate isomerase (*rpiB*), transketolase (*tktA*, *tktB*), transaldolase (*tal*), GAP dehydrogenase (*gapA*), phosphoglycerate kinase (*pgk*), phosphoglycerate mutase (*gpmI*), enolase (*eno*), pyruvate kinase (*pykA*), PEP carboxykinase (*pckA*), oxaloacetate decarboxylase (*oadA*,*oadB*), lactate dehydrogenase (*ldh*), pyruvate formate-lyase (*pfl*), pyruvate ferredoxin oxidoreductase (*pfor*), phosphate acetyltransferase (*pta*), acetate kinase (ack), malate dehydrogenase (*mdh*), fumarate hydratase (*fumB*), fumarate reductase (*frdA*, *frdB*), citrate synthase (*citZ*), aconitate hydratase (*acnA*), isocitrate dehydrogenase (*icd*), 2-oxoglutarate oxidoreductase (*korA*, *korB*), succinate-CoA ligase (*sucD*), propionyl-CoA:succinate CoA transferase (*scpC*), methylmalonyl-CoA mutase (*scpA*, *mutA*), methylmalonyl-CoA epimerase (*mce*), (S)-methylmalonyl-CoA decarboxylase (*mmdA*-*C*)

Subsequently, we analyzed the product spectrum of the species after 24 h of growth, except for *B. propionicifaciens* (sample at 45 h). At that timepoint, cells were already in stationary growth phase (Fig S1A, additional file 2) and glucose had been consumed completely (Fig S1B). The product concentrations of the cultures varied greatly between the species, although addition of yeast extract did not impact the product formation to a large extend (Tables 1 and S3, Additional file 2). The culture of *B. propionicifaciens* was the only one that exclusively produced propionate and acetate (12.7 and 6.1 mM). All other species also produced succinate in varying amounts (up to 7.8 mM). The highest propionate to succinate ratio in DMMG (2.1 mol_pro_/mol_suc_) was reached by *B. graminisolvens*. Formate could be detected in almost all species, although half of them produced only small amounts of less than 1 mM. It is surprising, that *B. graminisolvens* produced almost twice as much formate as acetate. Given the proposed route of formate production via PFOR and/or pyruvate-formate-lyase (PFL, Fig. 1), at least the same concentration of acetate would be expected [32]. Lactate was measured consistently only in cultures of *B. luti,* and butyrate could not be detected in any of the strains. The propionate yield varied among the strains. The highest value was reached by *B. propionicifaciens* with 0.39 g_pro_/g_gluc_ (DMMG-Y) and 0.34 g_pro_/g_gluc_ (DMMG), respectively. The molar ratio of propionate to acetate (2.0 mol_pro_/mol_ac_) corresponded to the suggested pathway (glycolysis, pyruvate oxidation by PFOR, and reduction of PEP to propionate via the succinate pathway, Fig. 2). It is slightly higher than the results of an earlier study, where a propionate/acetate ratio of about 1.5 was reached from glucose in a complex medium with hemin and vitamins (PYHVG) [33]. For comparison: *P. acidipropionici* reached a propionate/succinate ratio of 14.6 mol_pro_/mol_suc_ in a pH-controlled setup, while *P. freudenreichii* ssp*. shermanii* reached a ratio of 11.3 mol_pro_/mol_suc_. The propionate + succinate/acetate ratio was 1.7 and 1.4 mol_pro+suc_/mol_ac,_ respectively [34].

**Table 1:** Product formation of six *Bacteroides* strains

Products [mM]						
Yeast extract [ ±]	*B.* *ce.* CRE21	*B.* *gra*. XDT-1	*B.* *int.* 341	*B.* *luti* UasXn-3	*B. xylan. *XB1A	*B.* *pro.* SV434
Propionate						
−	3.1 ± 0.09	8.0 ± 0.40	3.2 ± 0.17	2.3 ± 0.05	2.4 ± 0.15	12.7 ± 0.50
+	3.6 ± 0.16	8.8 ± 0.15	3.8 ± 0.16	2.1 ± 0.44	2.8 ± 0.07	13.8 ± 0.32
Acetate						
−	8.0 ± 1.01	3.2 ± 0.12	8.4 ± 0.43	10.3 ± 0.77	8.1 ± 0.41	6.1 ± 0.21
+	7.9 ± 0.33	3.5 ± 0.35	8.5 ± 0.59	11.0 ± 0.71	8.4 ± 0.05	6.8 ± 0.22
Succinate						
−	2.7 ± 0.51	3.8 ± 0.29	3.1 ± 0.16	6.8 ± 0.19	4.4 ± 0.32	0.0 ± 0.00
+	3.3 ± 0.23	3.7 ± 0.31	3.2 ± 0.20	7.8 ± 0.94	5.0 ± 0.14	0.0 ± 0.00
Lactate						
−	0.4 ± 0.57	0.0 ± 0.00	0.1 ± 0.19	1.5 ± 0.46	0.0 ± 0.00	0.0 ± 0.00
+	0.0 ± 0.00	0.0 ± 0.00	0.2 ± 0.21	0.7 ± 0.92	0.0 ± 0.00	0.0 ± 0.00
Formate						
−	0.2 ± 0.22	5.9 ± 0.22	1.7 ± 0.96	4.7 ± 0.34	0.5 ± 0.13	0.0 ± 0.00
+	0.2 ± 0.06	6.4 ± 0.59	2.0 ± 0.93	5.1 ± 0.02	0.9 ± 0.31	1.0 ± 0.15
Yield [g_pro_/g_gluc_]						
−	0.09 ± 0.00	0.23 ± 0.01	0.09 ± 0.00	0.06 ± 0.00	0.07 ± 0.00	0.34 ± 0.00
+	0.10 ± 0.00	0.25 ± 0.01	0.11 ± 0.00	0.06 ± 0.01	0.08 ± 0.00	0.39 ± 0.01
Molar ratio [mol_pro_/mol_suc_)						
−	1.2	2.2	1.1	0.3	0.6	–
+	1.1	2.4	1.2	0.3	0.6	–
Molar ratio [(mol_pro_ + mol_suc_) /mol_ac_]						
−	0.7	3.7	0.8	0.9	0.8	2.1
+	0.9	3.7	0.8	0.9	0.9	2.0

Cells were grown in defined minimal medium with 15 mM glucose (DMMG), either with ( +) or without (−) 0.5 g/l yeast extract in 48-well plates with 0.5 ml culture volume under anoxic conditions (N_2_/CO_2_; 80/20; vol/vol). The cultures were incubated for 24 h (*B.* *propionicifaciens* for 48 h) at 30 °C (*B.* *propionicifaciens*) and 37 °C (all others). Samples for product formation were retrieved at the end and analysed via HPLC. Average values and standard deviation of three biological replicates are shown. *B. ce.*, *B. cellulosilyticus*; *B. gra., B. graminisolvens; B. int., B. intestinalis; B. luti, B.luti;B. xylan, B. xylanisolvens; B. pro., B. propionicifaciens*

*B. graminisolvens* cultures reached the second highest yield of 0.23 and 0.25 g_pro_/g_gluc,_ respectively, and a propionate/acetate ratio of 2.5 and 2.6 mol_pro_/mol_ac_. A similar ratio of 2.4 was found in a previous study, although there only 3.6 mM propionate from 55 mM of glucose (in PYHVG medium) were reported, and no production of formate [35]. Thus, different cultivation methods can increase the relative product formation independent of glucose concentrations.

Hence, *B. propionicifaciens* and *B. graminisolvens* were chosen for further examination of their propionate production route. Interestingly, *B. propionicifaciens* with the lowest growth rate, produced the highest amount of propionate and exhibited the best propionate yield of all the 10 species. *B. graminisolvens* reached a slightly lower yield but showed a relatively high growth rate even on defined medium. Since other products generated from acetyl-CoA, such as butyrate, ethanol or acetone, were not produced by *B. graminisolvens*, the unexpectedly high formate and low acetate concentrations indicate that the metabolism varied from the expected model in which acetyl-coenzyme A is completely converted into acetate, resulting in equal or higher amounts of acetate compared to formate. In addition, *B. graminisolvens* has been shown to utilize the polymers xylan, pectin and starch [35], which makes it an interesting candidate for further examination.

### Genomic evaluation of metabolic pathways

The two selected *Bacteroidia* species exhibited differential growth characteristics and product spectra. We, therefore, screened the genomes of these species for the most important genes of the glucose and energy metabolism, to gain a comparative overview of the metabolic pathways (Tables S4 and S5, additional file 3). The findings of our genomic evaluation are summarized in Fig. 2. The genes for the glycolysis were present in the genomes of both species. They also both carry the genes for the oxidative part of the pentose phosphate pathway encoding glucose-6-phosphate dehydrogenase, 6-phosphogluconolactonase and 6-phosphogluconate dehydrogenase, for the conversion of glucose to ribulose-5-phosphate, NADPH and CO_2_. While transketolases were found in both genomes, only *B. graminisolvens* harbored a transaldolase gene that is closely related to a transaldolase of *Bacillus subtilis.* Although this gene is annotated as fructose-6-P-aldolase, it has been shown for *P. vulgatus* to have transaldolase activity (BVU_3333) [21]. It is, therefore, possible, that both the oxidative and non-oxidative part of the pentose phosphate pathway is present in *B. graminisolvens. B. propionicifaciens* does not carry a transaldolase gene, but the pentose phosphate pathway might still be able to function via the pyrophosphate dependent fructose-6-P-kinase and fructose aldolase. The first enzyme phosphorylates sedoheptulose-7-phosphate and the second one cleaves sedoheptulose-1,7 bisphosphate to dihydroxyacetone phosphate and erythrose 4-phosphate. This pathway has been confirmed to be present in S*. copri* (PREVCOP_03899 and PREVCOP_06123) and many *Bacteroides* strains harbor the respective genes [21, 36]. The genomes of both species, *B. graminisolvens* and *B. propionicifaciens* carry genes encoding homologues to these two enzymes (Protein ID: WP_024996790.1; WP_018109477.1; WP_024997536.1; WP_025069472.1) *B. propionicifaciens* has been described to utilize the pentose arabinose, although xylose, which is metabolized through the PPP, is not utilized [33]. A different degradation pathway may be used for arabinose then xylose [37], or the species may be missing a xylose transporter and the enzymes for xylose conversion to xylulose-5-phosphate [38]. Therefore, it remains to be elucidated whether a functional pentose phosphate pathway exists in *B. propionicifaciens*.

Hexose and pentose metabolism lead to the production of PEP, which can either be converted to oxaloacetate via PEP carboxykinase, (PEP-CK) or to pyruvate via pyruvate kinase (PK) in both strains. From oxaloacetate, the genes for the reductive branch of the TCA leading to the production of succinate via oxaloacetate, malate and fumarate were found. Citrate synthase and the other genes of the oxidative branch of the TCA-cycle towards the production of succinyl-CoA could be identified in both species as well*. B. graminisolvens* also harbored a gene for the succinate-Coenzym A-ligase, which is either ATP or GTP dependent, suggesting that succinate production might also occur through the forward TCA-cycle similar to *B. thetaiotaomicron* [39, 40]. Although *B*. *propionicifaciens* only produced acetate and propionate, while *B*. *graminisolvens* also produced formate and lactate under certain conditions (Table 1), all necessary genes for the production of formate, acetate and lactate from pyruvate were present in both organisms, namely, pyruvate:formate lyase, pyruvate:ferredoxin oxidoreductase, phosphotransacetylase, acetate kinase and lactate dehydrogenase. Putatively, gene regulatory events or different affinities or other biochemical properties of the enzymes may explain the different relative product ratios in the strains.

Toward the understanding of redox and energy metabolism of both species, we searched for a variety of hydrogenase genes. When compared to a gene sequence of a [FeFe]-hydrogenase of *Clostridium pasteurianum*, no similarities were found in *B. propionicifaciens* and only very low similarity (*E* value of 0.016) in *B. graminisolvens*. In contrast, the known hydrogen producer *B. cellulosilyticus* [31] harbored a gene similar to the [FeFe]-type hydrogenase with an *E* value of 4 × 10^–130^ and a query cover of 97%.

To confirm these findings, hydrogen (H_2_) was measured in the headspace of cultures grown in DMMG with 15 mM or 30 mM glucose until stationary growth phase (Fig. S2). Cultures of *B. cellulosilyticus* and *S. copri* served as positive and negative controls, respectively [20, 31]. Small amounts of H_2_ were detected in all cultures as well as in the media control. No difference in H_2_ concentration was observed in cultures with 30 mM glucose compared to 15 mM. Thus, *B. graminisolvens* and *B. propionicifaciens* did not produce H_2_ during glucose fermentation, and the absence of true hydrogenases may disable these species to utilize protons as electron sink, unlike *B. cellulosilyticus*, which exhibited significantly greater H_2_ concentrations in the headspace. The wide distribution of [FeFe]-Type hydrogenases among *Bacteroidia*, which can produce H_2_ by oxidizing ferredoxin, has been shown previously [41], and we could confirm these findings, since 7 of our tested strains seem to harbor the respective gene (Table S4, additional file 3) This is also reflected in the relatively low (propionate + succinate)/acetate ratio of 0.74 mol/mol of *B. cellulosilyticus*. Since it can regenerate reduced ferredoxin through H_2_ production, less propionate and succinate need to be produced to achieve a closed redox balance.

Genes for the energy metabolisms identified were an Na^+^-gradient generating NADH-quinone reductase (Nqr), the Rnf complex and the cytochrome bd ubiquinol oxidase, which have been described previously for *Bacteroidia* [22, 29, 42]. In addition, *B. graminisolvens* harbored the genes for the NADH-quinone oxidoreductase without the subunits E, F and G, similar to the headless variant of this enzyme, that has been described before in *Bacteroidia* [20].

Overall, all genes for the succinate pathway that has been described for *Bacteroidia* [19, 20] were present and both strains contained a similar genetic equipment. However, a few genes were missing in *B. propionicifaciens* compared to *B. graminisolvens,* namely the transaldolase gene from the pentose phosphate pathway, the succinyl-CoA-ligase as part of the citric acid cycle and the NADH-quinone-oxidoreductase. Whether these genetic differences are the main cause for the difference in product spectra that were observed, or other factors, such as differential regulation of these genes, remain to be examined. From a biotechnological perspective, fewer bio-products are of high interest as downstream product separation is facilitated.

### CO_2_ dependence of B. graminisolvens and B. propionicifaciens

**Fig. 3:**
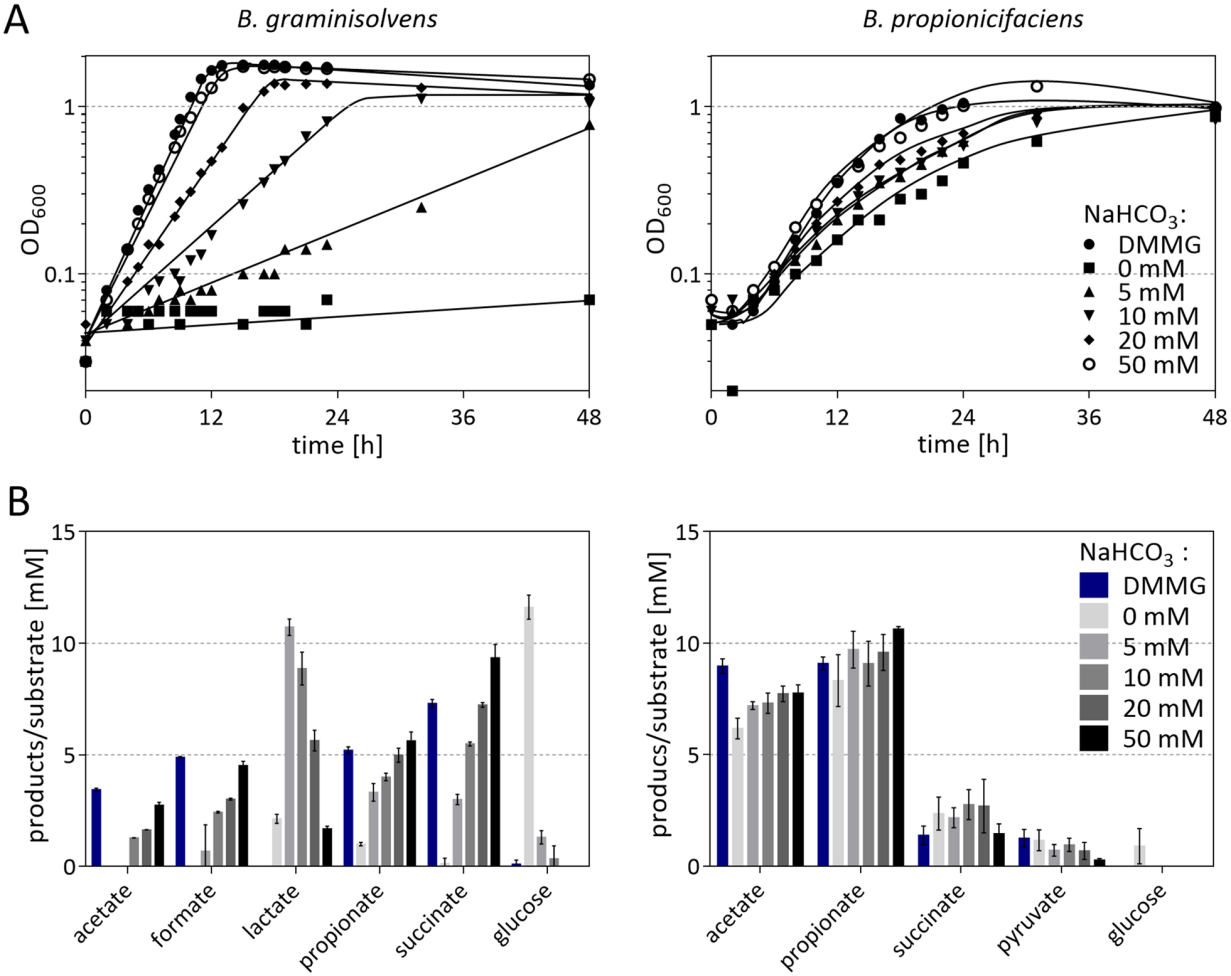
Influence of different NaHCO_3_ concentrations on growth (**A**) and product formation (**B**) of *B. graminisolvens* XDT-1 and *B. propionicifaciens* SV434. The experiment was performed in three biological replicates, with one representative shown (**A**). Cells were either cultured in DMMG or in a medium that was flushed with 100% N_2_ and contained a defined amount of NaHCO_3_. All cultures of *B. propionicifaciens* were supplemented with 0.5 g/l yeast extract to increase growth rate. HPLC-samples were taken at 48 h of incubation

It has been shown that growth of *Bacteroidia* is dependent on CO_2_ to a certain extent, since it is needed for PEP or pyruvate carboxylation to oxaloacetate [19, 20, 43]. In batch and continuous cultures of *B. fragilis*, low CO_2_ concentration, however, favored propionate over succinate formation [43], which is reflecting the subsequent decarboxylation of methylmalonyl-CoA to propionyl-CoA. To test the influence of varying amounts of CO_2_ on *B. graminisolvens* and *B. propionicifaciens*, growth experiments were performed, and product concentrations were measured in modified DMMG. It was flushed with N_2_ and contained defined amounts of NaHCO_3_ (releasing CO_2_), while conventional DMMG, containing 4 g/l NaHCO_3_ (47 mM) and flushed with N_2_/CO_2_ (80%/20%, vol/vol), was used as a control. To increase the growth rate of *B. propionicifaciens*, 0.5 g/l of yeast extract were added to the cultures. The growth rate of *B. propionicifaciens* slightly decreased from 0.19 h^−1^ in DMMG-Y to 0.13 h^−1^ without NaHCO_3_, but similar growth characteristics were observed in medium with 50 mM NaHCO_3_ compared to the control (Fig. 3A). The final OD_600_ was the same for all cultures. *B. graminisolvens* also showed comparable growth in the modified medium with 50 mM NaHCO_3_ (0.313 h^−1^) as in DMMG (0.336 h^−1^), but growth was significantly decelerated with decreasing NaHCO_3_ concentrations and almost stopped without NaHCO_3_. It is recommended to establish a CO_2_ concentration of at least 5% in the headspace for cultivation of *Bacteroides* [44]. We show here that this is not necessary for *B. propionicifaciens,* but CO_2_ addition does increase its growth rate. The growth behaviour of *B. graminisolvens* was comparable to *P. vulgatus,* for which no growth was detected with 0 mM [20]*.* The final OD_600_, increased rapidly with CO_2_ addition, so that half of the maximal OD_600_ was reached with 5 mM of NaHCO_3_. *S. copri* on the other hand, which is not able to produce propionate, could only grow at a minimal concentration of 10 mM NaHCO_3_. In *Propionibacterium acidipropionici*, CO_2_ addition slightly decreased final OD_600_ of the cultures, although product formation was only affected to a minimal extend. This was explained by CO_2_ generated during acetate production and the oxidative part of the oxPPP [45].

Cultures of *B. propionicifaciens* produced propionate, acetate, succinate and small amounts of pyruvate, with the propionate yields ranging between 0.25 to 0.31 g_pro_/g_gluc_ (Fig. 3B). Interestingly, the highest yield was reached in 50 mM NaHCO_3_ buffered cultures, whereas the DMMG-Y culture showed the lowest yield, however, with relative low variations between the different culture conditions. Product formation of *B. graminisolvens*, on the contrary, was strongly influenced by CO_2_. In correlation with decreased growth, all product concentrations decreased with lower NaHCO_3_ concentrations except for lactate, which was the main product in cultures with 5 mM NaHCO_3_ (10.7 mM). Accordingly, the propionate yield decreased from 0.15 g_pro_/g_gluc_ in DMMG to 0.11 g_pro_/g_gluc_ with 5 mM NaHCO_3_. However, the propionate/succinate ratio increased slightly from 0.7 in DMMG to 1.1 mol_pro_/mol_suc_ in 5 mM NaHCO_3_ medium, showing that low CO_2_ concentrations do not lead to a complete shift towards propionate production. In conclusion *B. propionicifaciens* is overall better suited to thrive at lower CO_2_ conditions than *B. graminisolvens*. In their metabolic pathway, CO_2_ is consumed for carboxylation of PEP to oxaloacetate but is released again by methlmalonyl-CoA-decarboxylase. In addition, CO_2_ can be released by the pyruvate-ferredoxin oxidoreductase during formation of acetyl-CoA. As formation of oxaloacetate is the first step in propionate production, CO_2_ needs to be present to form propionate. *B. propionicifaciens* showed a higher propionate to succinate ratio and, presumably needs less CO_2_ for this reason. It also does not produce formate by PFL, which means a net production of CO_2_ can be expected. Thus, *B. propionicifaciens* might create its own CO_2_ environment during growth. Low CO_2_ concentrations had a minor effect on product formation of *B. propionicifaciens* but led to a slight increase in the propionate to succinate ratio for *B. graminisolvens*, although the increasing amount of lactate in low CO_2_ conditions leads to an overall decrease of propionate yield. In the absence of CO_2_, *B. graminisolvens* was obviously not able to carboxylate PEP, and, therefore, must switch to the reduction of pyruvate to lactate to oxidize the NADH formed in glycolysis. This increase in lactate production in batch cultures with low CO_2_ concentrations was observed before in *B. fragilis* and *P. vulgatus* [43, 46]. Interestingly, propionate yields of both fermentations decreased in Hungate-tubes compared to plate-reader experiments. This might be a result of the increasing gas-pressure in the air-tight tubes compared to cultivation in an anaerobic tent, which provides a much bigger gaseous “headspace”. Raised CO_2_ levels can then induce the cells to release succinate into the medium instead of further converting it to propionate [47, 48]. High CO_2_ levels have also been shown to have an inhibitory effect on the PEP-CK [43]. Therefore, a large headspace or a possibility to exhaust excess CO_2_ should be provided for optimal propionate production.

### Sugar conversion to acids by cell suspension

**Fig. 4:**
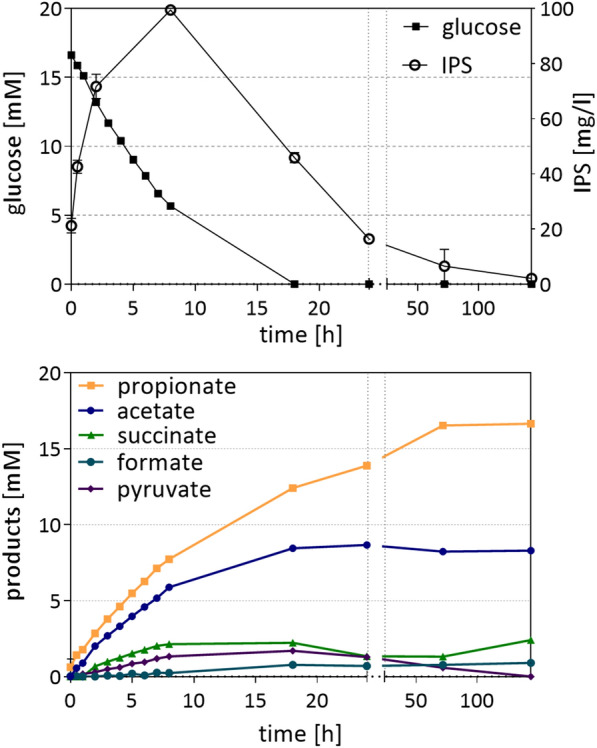
Glucose conversion and IPS in resting cells of *B. propionicifaciens* SV434*.* Three biological replicates (40 ml each) of resting cells (in RCB) with an average protein concentration of 0.46 mg/ml were incubated at 37 °C with 15 mM of glucose in serum flasks with N_2_/CO_2_ (80%/20%, vol/vol) in the headspace

Experiments with resting cells were performed to determine, whether the proposed metabolic model (Figs. 1 and 2) matched actual product formation from glucose. *B. propionicifaciens* produced an average of 16.0 ± 0.12 mM of propionate, 2.4 ± 0.04 mM succinate, 8.3 ± 0.06 mM acetate and 0.9 ± 0.01 mM formate from 16.5 mM of glucose (Fig. 4). Up to 1.6 ± 0.03 mM pyruvate was also detected during fermentation, but concentrations decreased towards the end, when glucose was no longer available. Since no growth occurred due to the absence of nutrient media compounds, they produced more acids compared to growth experiments, as the metabolites were not branched off for the production of cell mass. Interestingly, product ratios changed slightly compared to growth experiments. While propionate and succinate production increased by around 4 mM and 2 mM, respectively, acetate concentration did not change. In consequence, the propionate + succinate/acetate ratio increased to 2.2 ± 0.06 mol/mol. The increase in reduced products in cell suspensions in comparison to that in growth experiments may be explained by the fact that cell mass of *Bacteroidia* is more reduced than the substrate glucose (C_1_H_2_O_1_). Analysis of *S. copri* cells revealed an elemental composition of C_1_H_1_._79_O_0.44_ [20] and similar results were also obtained for *B. propionicifaciens* (C_1_H_1.85_O_0.54_) and *B. graminisolvens* (C_1_H_1.81_O_0.49_**)** through this study.

Intracellular polysaccharides (IPS) were also determined, as *Bacteroidia* are reported to produce glycogen or other intracellular glucose polymers [47, 49]. We found production of 99.4 ± 1.0 mg/l IPS during the course of the fermentation, which accounts for around 0.5 mM of glucose. The polysaccharide was produced when high glucose concentrations were present and degraded, when glucose concentrations fell below 5 mM. This observation is congruent to the increase of propionate from 11.8 ± 0.07 mM at 18 h of incubation to 15.9 ± 0.11 mM at 72 h of incubation, when glucose was no longer available, although glycogen usage alone would not be sufficient to explain the propionate increase. In contrast to our findings, it has been reported for cell suspensions of *B. thetaiotaomicron* to incorporate between 50% and 80% of glucose into IPS [47].

Product concentrations at 24 h of incubation were chosen for the calculation of redox and carbon balances, as concentrations of IPS were nearly the same for this timepoint as in the beginning. Calculation of the balances was based on the reaction equations from Table 2, which were set up according to the proposed model and genes present in the organisms. The carbon mass balance was calculated under the assumption that 1 glucose (1 × C6) is converted to 2 glyceraldehyde 3-phosphate (GAP) in glycolysis, and all products derived from the C3 carbon compound GAP in a 1:1 ratio (e.g., 1 GAP → 1 PEP, 1 PEP + CO_2_ → 1 oxaloacetate → 1 succinate → 1 propionate + 1 CO_2_; Table 2). According to the calculations from Table 3, only 74% of the consumed carbon could be recovered in the measured products. With the assumption that only this amount of glucose was actually converted into products, 87% of produced reduction equivalents [H] were retrieved, representing a nearly closed redox balance. Using the same calculation with the product concentrations at 144 h of incubation resulted in a GAP carbon conversion of 72% and a [H] yield of 100%

**Table 2:** Reaction equations for the calculation of carbon and redox balances in *Bacteroidia* according to proposed fermentation models

Glycolysis					
1	1 glucose		=	2 GAP	
oxPPP					
	3 glucose	+ 6 NADP^+^	=	5 GAP	+ 6 (NADPH + H^+^)
2	1 glucose	+ 2 NADP^+^	=	$\frac{5}{3}$ GAP	+ 2 (NADPH + H^+^)
3	1 GAP	+ 1 NAD^+^	=	1 PEP	+ 1 (NADH + H^+^)
Products					
4	1 PEP		=	1 pyruvate	
5	1 PEP	+ 2 (NADH + H^+^) + CO_2_	=	1 succinate + 2 NAD^+^	
6	1 PEP	+ 2 (NADH + H^+^)	=	1 propionate + 2 NAD^+^	
7	1 PEP	+ 1 Fd_ox_	=	1 acetate + 1 Fd_red._ + CO_2_	
8	1 PEP		=	1 acetate + 1 formate	
9	1 PEP	+ 1 (NADH + H^+^)	=	1 lactate + 1 NAD^+^	

ATP generation was not included. Glyceraldehyde-3-Phosphate (GAP), Phosphoenolpyruvate (PEP), oxidative pentose phosphate pathway (oxPPP)

**Table 3:** Calculation of redox and carbon balances of resting cells of *B. propionicifaciens*

		A	B	C
		Substrate/products [mM]	[H]	GAP
			[mM]	[mM]
Glucose				
1	Glycolysis→GAP	16.6		33.2
2	oxPPP→GAP	–	–	–
3	GAP→PEP	33.2	33.2	
Products				
4	pyruvate	1.2	0.0	1.2
5	succinate	1.3	−2.7	1.3
6	propionate	13.3	−26.6	13.3
7	acetate	7.9	7.9	7.9
8	acetate + formate	0.7	0.0	0.7
9	lactate	0.0	0.0	0.0
	GAP _products_ [mM]			24.4
	GAP yield [%]			74
	[H_total_] [mM]		24.4	
	[H_products_] [mM]		−21.3	
	[H] yield [%]		87.1	

Product concentrations after 24 h of incubation were utilized for calculations according to Table 3. NADH + H^+^, NADPH + H^+^ and Fd_red_. were simplified as [H]. Oxidation reactions of the substrate received a positive value, whereas reduction reactions received a negative value. GAP_products_: sum of C4—C9. GAP yield: GAP_products_ *100/A3. [H_total_]: reduction equivalents produced from GAP _products_ + B2. [H_products_]: sum of B4—B9, the reduction equivalents used for the formation of the fermentation products. [H] yield: [H_products_] *100/[H_total_]

**Fig. 5:**
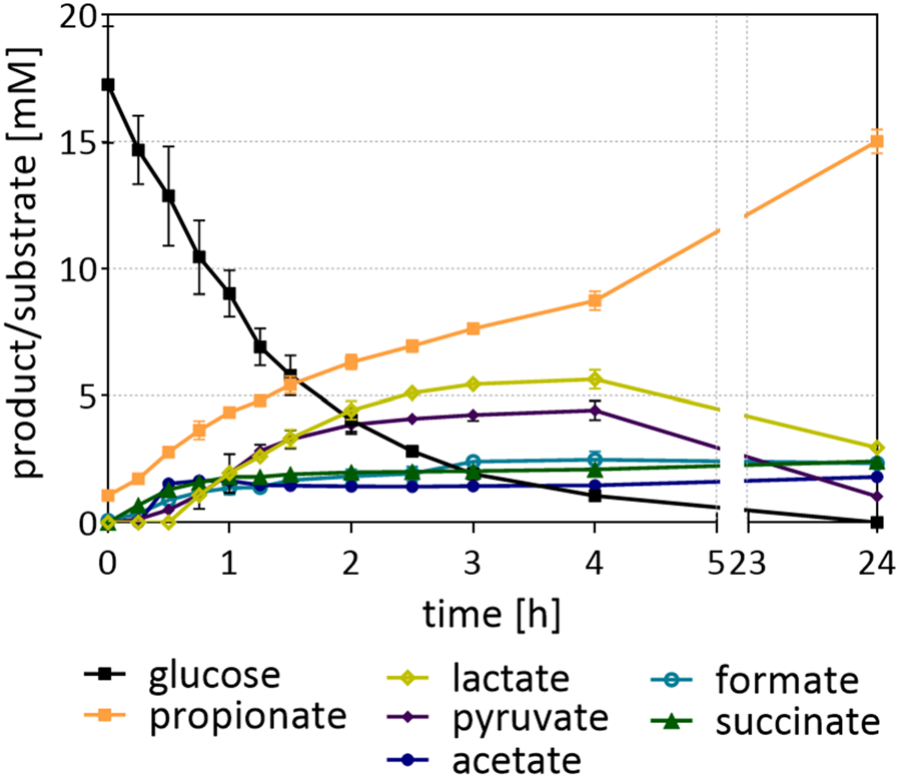
Glucose conversion by resting cells of *B.* *graminisolvens* XDT-1*.* 20 ml of resting cells (in RCB) with a protein concentration of 0.54 mg/ml were incubated at 37 °C with 15 mM of glucose in serum flasks with N_2_/CO_2_ (80%/20%, vol/vol) in the Headspace. Glucose and product concentrations were measured by HPLC. Average and standard deviation of three cultures are shown (*n* = 3 ± SD)

The experiment was also carried out with *B. graminisolvens* cultures, which showed significantly different product ratios compared to the DMMG growth experiments (Fig. 5). 17.25 mM glucose was consumed at a rate of 5.11 mM per hour. While propionate was still the most abundant acid (13.9 mM ± 0.4 mM), lactate and pyruvate were measured in concentrations of up to 5.6 ± 0.3 mM and 4.4 ± 0.3 mM, respectively. Both were reabsorbed towards the end of cultivation, indicating a rate limitation either in the PEP carboxylation (PEP-CK) or pyruvate oxidation by PFOR. Acetate, formate and succinate were produced in similar concentrations between 1.8 and 2.3 mM. This change of product ratio is similar to the one observed under the CO_2_-limitation. When carbon and redox balances were calculated, assuming that glucose is metabolised solely through glycolysis, 66% of GAP was retrieved but 157% of [H] meaning that the amount of GAP from glucose consumption through glycolysis is not sufficient to produce the amount of reduction equivalents needed for the generation of the fermentation products. Therefore, we assumed that 37% of the glucose are metabolized through the oxidative pentose-phosphate pathway. Theoretically, this resulted in a GAP yield of 70% and a [H] yield of 101%.

Overall, these measurements show that resting cells of both species produced significant amounts of propionate from glucose, which may even exceed the theoretical propionate + succinate to acetate ratios. Hence, the results leave open questions regarding the metabolic pathways of the two species. We demonstrated production of storage polysaccharides in *B. propionicifaciens*. However, they were not sufficient to explain the intermediate low carbon recovery at 24 h. Since the carbon and electron balance for *B. propionicifaciens* was nearly closed at 144 h, we assume the accumulation of a yet undetected intracellular or extracellular intermediate, that was consumed between 24 and 144 h of incubation. *B. graminisolvens* relies on the fermentation of glucose partly through the oxPPP. To which extend glucose is metabolized in both pathways has not been characterized for these strains. A study of *B. ovatus* has revealed a correlation of the product spectrum to carbon-levels. When carbon was in excess, more succinate was produced, while carbon limited conditions favored propionate production, as this can recycle CO_2_ for further succinate formation [48]. This was also observed in *B. thetaiotaomicron*, where propionate formation was enhanced with lower growth rates in glucose limited continuous culture [50].

### pH-controlled cultivation

**Fig. 6:**
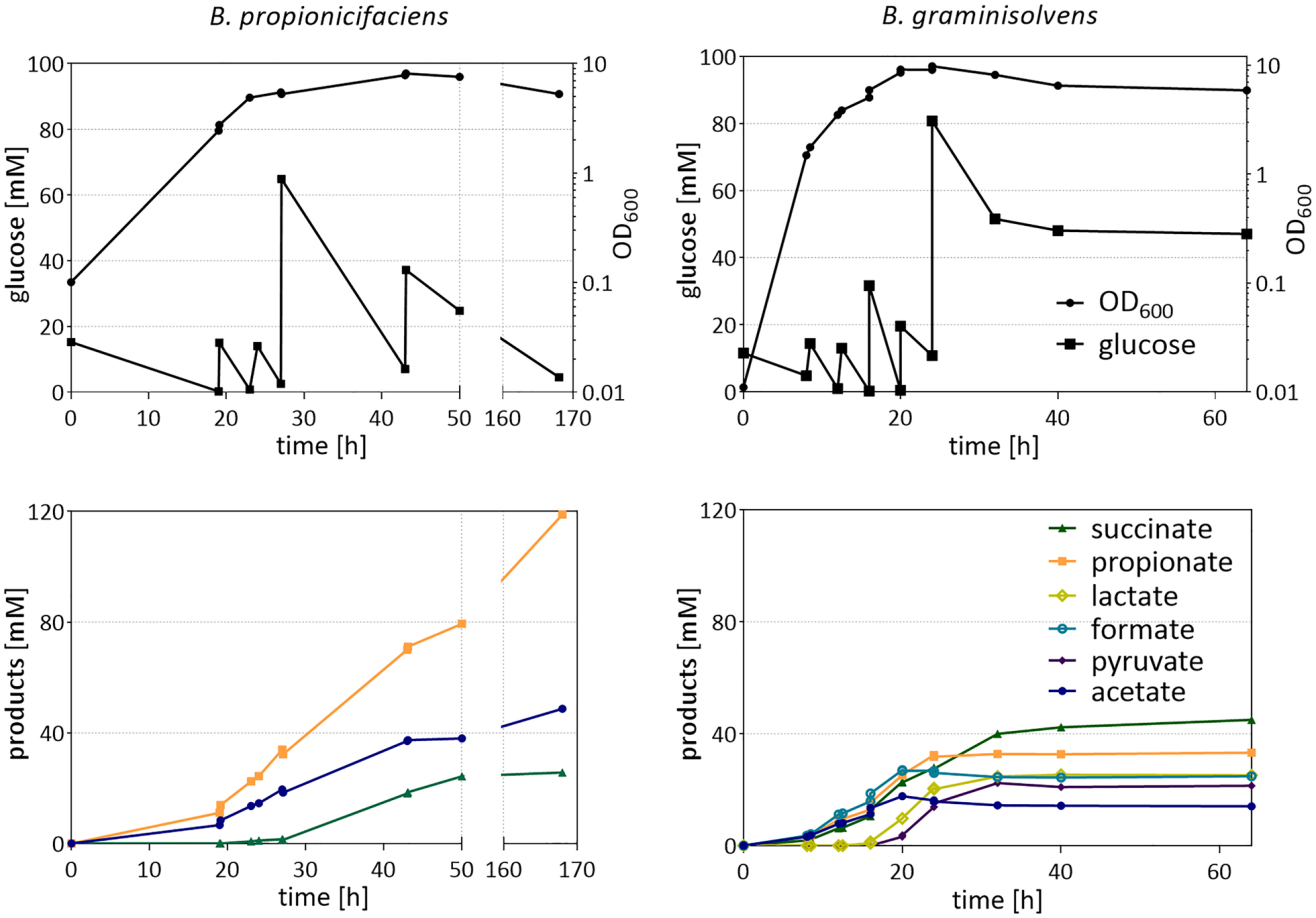
Growth, glucose concentration and product formation of *B. propionicifaciens* SV434 (30 °C) and *B. graminisolvens* XDT-1 (37 °C) in a pH-controlled fed-batch cultivation. DMMG-Y with 1 g/l of yeast extract was used, with twice the amount of NH_4_Cl, trace elements and vitamins. A minimal pH of 7 was maintained by automatic addition of 2 M KOH solution. The culture vessel was flushed with N_2_/CO_2_ (80%/20%, vol/vol) at a rate of 1 l/h

To increase propionate titers, we cultivated the cells under constant pH in DMMG-Y with 1 g/l yeast extract (Fig. 6). In addition, since high glucose concentrations have been shown to increase the amount of side products, glucose was fed to the medium in small pulses between 15 and 60 mM at a time.

In these conditions, *B. propionicifaciens* grew exponentially until 23 h of incubation to an OD_600_ of 4.9. The growth then slowed down until it reached its maximal OD_600_ of 7.8 at 43 h of incubation. Overall, the culture consumed 130 mM (23.4 g/l) of glucose while producing 119 mM (9.3 g/l) propionate, 49 mM (2.9 g/l) acetate and 26 mM (3.1 g/l) succinate. After the first two pulses of 15 mM of glucose, succinate was not detected in the supernatant, but started appearing in higher amounts after 60 mM of glucose were added at once. Succinate concentration increased less after the pulse of 30 mM at 43 h. We calculated a propionate titre of 8.8 g/l with a propionate yield of 0.9 mol_pro_/mol_gluc_ or 0.37 g_pro_/g_gluc_. The overall productivity was 0.09 g/l*h, with the highest productivity reached at 27 h with 0.21 g/l*h.

*B. graminisolvens* grew faster, as it reached its highest OD_600_ of 9.8 at 24 h of incubation. It received 160 mM of glucose from which it consumed 107 mM (19.26 g/l) and produced 14 mM (0.8 g/l) acetate, 25 mM (1.2 g/l) formate, 25 mM (2.2 g/l) lactate, 33 mM (2.4 g/l) propionate and 45 mM (5.3 g/l) succinate, which corresponds to a yield of 0.31 mol_pro_/mol_gluc_ or 0.13 g_pro_/g_gluc_. During the first 16 h of cultivation, propionate, succinate, acetate and formate were produced in similar amounts, while pyruvate and lactate only started to appear after the addition of 30 mM of glucose. With further cultivation, succinate became the main fermentation product. This could again be a symptom of carbon excess previously discussed [48], although other explanations are possible. In propionibacteria, the succinyl-CoA-transferase is discussed to be the limiting enzyme in the conversion of succinate to propionate [51], but little is known on the limitations in *Bacteroidia.* Glucose consumption stopped between 32 and 40 h and product concentrations did not change further from that timepoint. Addition of ammonia, vitamins and trace elements to the media at 24 h had no effect on growth or product formation, showing that nutrient availability was not the reason the cells stopped metabolizing glucose. There have been previous studies with *Bacteroidia* grown in pH-controlled fermentations, but none have focused on propionate production so far. For example, *Segatella copri* grew in a similar setup with complex medium to an OD_600_ of 11 and consumed around 300 mM (54 g/l) of glucose before product formation ceased. However, propionate and lactate were not produced by this strain, thereby a similar change of product ratios could not be detected [52]. In *P. vulgatus,* a pH-controlled setup in DMMG without buffer components produced less lactate than cultures with additional buffer [53]. In a similar pH-controlled setup, *P. acidipropionici* consumed up to 40 g/l of glucose and produced 16.3 g/l propionate [45], almost twice the amount consumed by *B. propionicifaciens,* while the yield (0.41 g_pro_/g_g__luc_) was comparable to the 0.37 g_pro_/g_g__luc_ of *B. propionicifaciens*. The propionate/succinate ratio of 11 mol_pro_/mol_suc_ also exceeded the ratio reached in our setup (4.6 mol_pro_/mol_suc_). Another study with *P. acidipropionici*, using immobilized cells and whey lactose as substrate, reported propionate titers well above 100 g/l [54]. In addition, the use of various pretreated agricultural wastes has been explored, as well as different approaches in process optimization [6]. Still, no commercial propionate production has been installed to our knowledge. While the tested *Bacteroidia* lag in yield and product ratios compared to the well-studied propionibacteria, they still hold many opportunities for optimization. The advance in genetic tools for example: with a markerless deletion method already established in *P. vulgatus* [26] side product formation of *B. graminisolvens* can be reduced. Further physiological insights will help to find out, why *B. propionicifaciens* has a more effective propionate conversion, which can then be applied to species with a broader substrate spectrum. Furthermore, it has been shown in bioreactor studies with *B. fragilis*, that changes of media composition, inoculum size and cultivation conditions increased its succinate production by more than tenfold [55]. Therefore, process optimization might further increase the propionate production of the two tested strains.

## Conclusions

Although many studies focus on *Bacteroidia* for their role in gut health, little is known about their propionate formation, especially in lately isolated strains or described species. We, therefore, started with an overview of strains that produce propionate, showing that this trait is widespread among this group. Ten species were selected from this list and grown under defined conditions to compare growth and propionate production on glucose. We saw great variability to their growth, as some strains were dependent on contents from yeast extract, while it showed no effect in others. In addition, product spectra varied greatly. We then chose the two best propionate producing strains and showed that they produce different products, although their core metabolic genes are mostly the same. Most striking is that the production of lactate was only observed in *B. graminisolvens* but not in *B. propionicifaciens*, although both organisms seem to harbor the lactate dehydrogenase gene. Growth and product formation in low CO_2_ conditions showed that *B. propionicifaciens* was less affected than *B. graminisolvens*, which showed altered product spectra and almost no growth without CO_2_. Carbon mass and electron balances from resting cell experiments were almost closed under the assumption that part of sugar carbon had been oxidized by the oxPPP. The pH-controlled fed-batch showed the potential of *B. propionicifaciens* to produce propionate at relatively high yields and titers. While our fermentation result do not match the yield, or productivity reached by propionibacteria, we anticipate that by optimization of the culture conditions and future genetic engineering approaches succinate, lactate and formate production will be significantly reduced while the propionate titer will increase. Overall, with further efforts to investigate their propionate, succinate and prebiotics production, polymer degradation and the recent development of genetic tools, *Bacteroidia* have the potential to become production platform strains for conversion of plant-derived polymers to bioproducts.

## Methods

### Overview of propionate producing Bacteroidia strains

To select *Bacteroidia* species for this study, a literature search was performed by searching for the term “Bacteroidetes” in combination with “propionate” or “propionic acid” in pubmed (https://pubmed.ncbi.nlm.nih.gov/), which yielded 656 entries in total. Title and abstract of the entries were manually scanned for information on propionate production. In addition, “Cited articles” and “Cited by” section of selected entries were searched for more strains. The search focused on description of new strains or studies on single species rather than propionate forming consortia, and only mesophilic strains, for which propionate production from sugars or carbohydrate polymers was measured, were included in the overview. Isolates from oral origin were also not considered, as this study focused on gut associated *Bacteroidia.*

### Strain selection and cultivation techniques

Ten strains from the list in Table S1 (additional file 1) (written in bold letters) were selected to be further examined in this study. The strains were classified as risk Group 1 according to the German technical rule for biological agents (TRBA) from 2015. We then chose strains that either were described to produce propionate as their main fermentation product and/or utilized a wide variety of complex substrates. *Phocaeicola vulgatus*, *Parabacteroides johnsonii* and *Bacteroides cellulosilyticus* as well as the propionate negative strain *Segatella copri* DSM 18205 (previously *Prevotella copri* [30]), were provided by Prof. Uwe Deppenmeier, University of Bonn (Institute of Microbiology and Biotechnology, University of Bonn, 53115 Bonn, Germany). All other strains were obtained through the “German Collection of Microorganisms and Cell Cultures GmbH” (DSMZ). Unless otherwise stated, strains were grown in defined minimal medium with 15 mM glucose (DMMG). It is based on the minimal growth requirements of *B. fragilis* [56] and was modified by Franke et al. [20]. It contained per liter: NaHCO_3_ (4 g) L-Cystein-HCL (0.5 g), KH_2_PO_4_ (1.7 g), K_2_HPO_4_ (2.2 g) NaCl (0.9 g), NH_4_Cl (0.76 g) MgCl_2_ × 6 H_2_O (0.1 g) CaCl_2_ × 2 H_2_O (26 mg), FeSO_4_ (2.78 mg), SL-6 solution (2 ml) (Table S6, additional file 4) and 0,1% Resazurin (1 ml). The pH was adjusted to 8.0 with KOH and the medium was aliquoted into glass anaerobic culture tubes (Hungate tubes) or serum flasks. These were flushed with a N_2_/CO_2_ gas mixture (80%/20% vol/vol), and subsequently sealed with butyl rubber seals prior to autoclaving. Glucose (15 mM), hemin (1 mg/l), vitamin K (1 µl/l) and 5 × Wolin solution with B_12_ (2 ml/l) (Table S7, additional file 4) were added to the medium from anoxic stocks before inoculation. *B. propionicifaciens* and *P. paurosaccharolyticus* were grown at 30 °C, all other strains were grown at 37 °C. A vinyl anaerobic chamber (Coy Laboratory Products, Grass Lake, USA) was utilized for anaerobic handling of cultures outside of gas tight flasks. It was operated with N_2_/CO_2_ (80%/20% vol/vol) and up to 4% H_2_.

### Growth and product screening of 10 Bacteroidetes strains

Growth experiments for species comparison were performed in an Epoch™ 2 microplate spectrophotometer (BioTek instruments, Agilent, Santa Clara, USA), which was set up in a vinyl-anaerobic tent (Coy-Laboratory Products, Grass Lake, USA). Cultures were grown either on DMMG or with an addition of 0.5 g/l yeast extract (DMMG-Y). They were first inoculated in Hungate tubes to an OD_600_ of 0.02 from a DMMG overnight culture and then transferred to the anaerobic tent. 500 µl of culture were distributed in two wells of 48-Well-Plates each. The plate reader was programmed to run measurement cycles every 15 min with one cycle consisting of 5 min of orbital shaking (282 cpm), 20 s of linear shaking (567 cpm) and measurement of the OD_600_. Samples for product measurements were taken at 0 h from the starting culture and at 24 h or 45 h. Experiments were performed in triplicates.

### Genetic analysis

To gain an overview of the metabolic and energetic pathways of the selected strains, a BLAST^®^ search [57] was performed. Amino acid sequences of characterized proteins were either obtained through UniProt (uniprot.org) or the proteindatabank of Europe (ebi.ac.uk/pdbe). First, a BLASTp search was performed with the non-redundant-protein sequences database for the ten species selected in this study as well as the well characterized species *Segatella copri* [20] for comparison. When no significant match was shown for a species, a BLASTn search was performed for the type strain of a species, using the whole genome shotgun database (wgs). Source of the query sequences and search parameters are listed in Table S4. To obtain strain specific data, a second BLASTn search was performed with two full genomes of each type-strain against the same amino acid sequences selected before (Table S5).

### Growth with varying amounts of NaHCO3

To test the CO_2_ dependency of *B. propionicifaciens* and *B. graminisolvens* as well as the influence of CO_2_ on product formation, growth experiments were performed in 10 ml DMMG in Hungate-tubes with varying amounts of NaHCO_3_. The medium was prepared with additional 50 mM of MOPS buffer and was flushed with 100% N_2_ instead of N_2_/CO_2_. For *B. propionicifaciens* cultures, 0.5 g/l yeast extract was added to increase growth rate (DMMG-Y). Cultures were inoculated from precultures grown on DMMG to an OD_600_ of 0.02, and incubated for 48 h. Growth was monitored by measurement of the OD_600_ and samples for HPLC analysis were taken at the beginning and the end.

### Preparation of resting cells

To prepare resting cells, 1000 ml *B. graminisolvens* or 3000 ml *B. propionicifaciens* cultures grown in DMMG or DMMG-Y, respectively, were harvested in mid to late exponential growth phase. To keep cell viable, an anoxic environment was maintained throughout the whole concentration process and experiment. The cells were then pelleted (8000 × *g*, 4 °C, 20 min) and washed twice in one fifth volume of resting cells buffer (RCB). It contained per litre: 4 g NaHCO_3_, 0.5 g L-cystein-HCL, 1.7 g KH_2_PO_4_, 2.2 g K_2_HPO_4_, 0.9 NaCl and 0.1% Resazurin (1 ml). RCB was prepared like DMMG but without glucose, hemin and the two vitamin solutions. The cells were then resuspended in either 100 ml (*B. graminisolvens*) or 200 ml (*B. propionicifaciens*) RCB and filled into airtight serum flasks and Hungate tubes. To eliminate the remaining H_2_ from the environment of the anaerobic tent, the headspace of the cultures was flushed with N_2_/CO_2_ (80%/20% vol/vol) for 5 min. Cultures were then incubated for 30 min at their respective growth temperatures to ensure that all remaining nutrients from the medium, supporting growth, were consumed. Next, glucose was added to a concentration of 15 mM and samples were taken for HPLC analysis and protein quantification. Cultures were then incubated at their respective growth temperature and samples for HPLC measurements were taken regularly. Another sample for protein quantification was retrieved at the end of the incubation period, to confirm that no cell growth had occurred. In resting cells of *B. propionicifaciens*, samples for glycogen quantification were taken in addition.

### pH controlled fed-batch cultivation

1.5 l fermentation-medium was prepared similar to DMMG but with twice the amounts of NH_4_Cl, MgCl_2_ × 6 H_2_O, CaCl_2_ × 2 H_2_O, FeSO_4_, SL-6 solution, hemin, vitamin K and 5 × Wolin solution with B_12_ as well as 1 g/l yeast extract to prevent growth inhibition due to limitations of these nutrients. L-cystein HCL was added to the medium after autoclaving from a sterile and anoxic 2.5 M stock solution. The medium was flushed with sterile gas mix after autoclaving and a constant flowrate of 1 l/h was maintained throughout the cultivation. 15 mM glucose were initially added to the medium and it was inoculated with 15 ml overnight culture in DMMG. The culture vessel was connected to a Biostat^®^ B plus controller system (Satorius, Göttingen, Germany) that maintained a constant pH of 7.0 with 2 M KOH solution and stirred the culture at 50 rpm. OD_600_, product formation and glucose concentration were measured regularly. When the glucose concentration went below 5 mM, 15 to 60 mM glucose were added to the culture. A decrease in OD_600_ was measured in the culture of *B. graminisolvens* after 24 h of incubation. Therefore, hemin, vitamin K, Wolin solution with B_12_, SL6-solution, FeSO_4_ and NH_4_Cl were added to the medium again in twice the amount contained in DMMG.

### Analytical methods

Growth was measured as optical density at 600 nm (OD_600_). Glucose and organic acids were quantified by HPLC. Samples were prepared by centrifugation (6000 × *g*, 4 °C, 20 min). Then, 250 µl of culture supernatant were acidified with 10 µl of 50% sulfuric acid (vol/vol) and centrifuged again (18000 × *g*, 4 °C, 5 min). A Shimadzu HPLC-system (Kyoto, Japan) equipped with SIL-20AC autosampler, LC-20AD pump and CTO-20AC column oven with organic-acid resin column (300 × 8 mm, Chromatografie Service GmbH, Langerwehe, Germany) was used with 5 mM sulfuric acid as liquid phase at a flow rate of 0.6 ml/min at 30 °C. Compounds were either detected with a refractive index detector or a UV–Vis detector set to 210 nm.

An enzyme assay containing hexokinase and glucose-6-phosphate dehydrogenase (Version 05, Roche, Grenzach-Wyhlen, Germany) was used to determine glucose concentrations during the fed-batch experiments. The assay was carried out according to manufacturer’s manual either with 10 µl of pure supernatant or with 1:2 and 1:5 dilutions in *A. dest*.

H_2_ was measured in the headspace of cultures in stationary growth phase grown in DMMG with either 15 mM or 30 mM glucose. A 1 ml sample was taken from the headspace using a gas tight syringe and directly applied to a gas chromatograph (8860 GC system, Agilent, Santa Clara, USA) equipped with a carboBOND column (50 m × 530 µm × 5 µm, Agilent, Santa Clara, USA). N_2_ was used as carrier gas at a flow rate of 5 ml/min and 50 °C. Gas content was detected with thermal conductivity detector operated at 180 °C.

A biuret reaction assay was performed for the quantification of protein in whole cells. [58] A pellet from 1 ml of culture was resuspended in 1 ml of distilled water. Then 125 µl of a 4 mM NaOH solution was added for lysis (99 °C, 10 min), followed by 5 min on ice. Next, 400 µl of copper-solution was added (potassium sodium tartrate (16.92 g/l), CuSO_4_ × 5 H_2_O (2.5 g/l) potassium iodide (6.24 g/l) and NaOH (10 g/l). Substances were dissolved separately and then mixed in above order. Samples were incubated for 30 min at 37 °C. A centrifugation step was added (13000 × *g*, 4 °C, 5 min) to remove the precipitate and absorption of the supernatant was measured at 546 nm. An analogous sample containing only *A. dest* was used as blank. Concentration was determined using a calibration with 1 ml of a standard solution containing bovine serum albumin.

For quantification of IPS such as glycogen, a colorimetric method using anthron was utilized [59]. For this, a pellet from 2.5 ml cell suspension was washed with 1 ml of *A. dest* and resuspended in 300 µl. Then, 200 µl of a Na_2_SO_4_ solution (2%, w/v) was added, followed by lysis at 70 °C for 10 min. Samples were then cooled on ice and 1 ml of methanol (99%, vol/vol) was added. Cells were centrifuged (12.000 × *g*, 4 °C, 3 min) and the supernatant was discarded as the pellet was washed in 1 ml of methanol for a second time. Next, 5 ml of freshly prepared anthron solution were added, incubated for 15 min at 90 °C and then cooled on ice. The extinction of the solution was measured at 620 nm against a pure anthron solution. 300 ml of a standard solution containing between 0 mg/l to 800 mg/l glycogen were prepared accordingly for calibration. The anthron solution contained per litre: H_2_SO_4_ (vol/vol, 750 ml), ethanol (96%, vol/vol, 50 ml), anthron (2 g). To estimate the molar amount of glucose stored in the glycogen, samples with up to 1000 mg/l of glycogen used for the standard solution, were hydrolysed in 0.9 M hydrochloric acid at 99 °C for 2 h. The glucose concentration was then measured via HPLC [60].

Cells for elemental analysis were grown in 500 ml DMMG in anoxic culture flasks, harvested during late exponential growth phase and washed with 100 ml *A. dest*. After freeze drying for at least 24 h, they were homogenized with mortar and pestle. The analysis was performed by the Fraunhofer Institute for Applied Polymer Research IAP (Potsdam, Germany).

### Supplementary Information

Additional file 1: Table S1. Selection of *Bacteroidia* that produce propionate and respective plant polymers that can be utilized. Acids are listed in decreasing order according to relative amounts produced. Strains used in this study are written in bold. Abbreviations: acetate (A), propionate (P), succinate (S); lactate (L); malate (M), butyrate (B); isobutyrate (IB); isovalerate (IV), fumarate (Fu), not determined (Nd).

Additional file 2: Table S2. Growth parameters of ten strains of the class *Bacteroidia*. Growth was determined by measurement of OD_600_ every 15 minutes for 24 h (45 h in case of *B. propionicifaciens*) in defined minimal medium with 15 mM glucose (DMMG) without yeast extract (-) or with 0.5 g/l yeast extract (+). The experiment was carried out in a plate reader set to 30°C or 37°C, respectively, placed in an anoxic chamber (Coy laboratory products, Grass Lake, USA). Mean values and standard deviation of three biological replicates are shown. OD_max_ was the highest OD_600_ value measured. The specific growth rates were determined from 5 consecutive timepoints by a regression analysis. Table S3. Product formation of two strains of the genus *Parabacteroides* and *Phocaeicola*. Cells were grown for 24 h (48 h for *B. propionicifaciens*) in defined minimal medium with 15 mM glucose (DMMG), either with (+) or without (-) 0.5 g/l yeast extract. Samples for product formation were retrieved at the end and analysed via HPLC. Average values and standard deviation of three biological replicates are shown. Fig. S1. Growth and remaining glucose concentration of 10 *Bacteroidia* cultures in DMMG with 15 mM glucose. (A) OD_600_ was measured every 15 minutes with a plate reader for up to 45 h (every second measurement shown). One representative curve of three is shown. (B) Glucose concentration after 24 h of cultivation. A second measurement was performed with *B. propionicifaciens* cultures after 45 h. Average and standard deviation of three biological replicates are shown. Fig. S2. Gas content in the headspace of *B. graminisolvens*, *B. propionicifaciens*, *S. copri* and *B. cellulosilyticus.* Cells were grown in defined minimal medium with either 15 mM (turquoise) or 30 mM of glucose (black) until stationary growth phase. Samples of the control gases and the headspace of the medium control are depicted in blue.

Additional file 3: Table S4. Results of BLASTp searches with 11 *Bacteroidia* species against amino acid sequences of characterized enzymes of the central carbon and energy metabolism. Table S5. Results of BLASTn searches with two genomes each of the strains *B. graminisolvens* XDT-1 and *B. propionicifaciens* SV434 against amino acid sequences of characterized enzymes of the central carbon and energy metabolism.

Additional file 4: Table S6. Components of SL-6 trace element solution per litre. Table S7. Components of 5 x Wolin solution per litre.

Additional file 5: Original datasets belonging to figures 3–6, table 1, table S2 and table S2.

Additional file 6: Original datasets belonging to table S2.

## Data Availability

The datasets supporting the conclusions of this article are included within the article and its additional files.

## Abbreviations

Ac: Acetate
BLAST: Basic logical alignment search tool
CAP: Cellulose acetate propionate
cpm: Cycles per minute
DHAP: Dihydroxyacetone-phosphate
DMMG: Defined minimal medium with glucose
DMMG-Y: Defined minimal medium with glucose and yeast extract
DSMZ: German Collection of Microorganisms and Cell Cultures GmbH
E4P: Eythrose 4-phosphate
F1,6BP: Fructose 1,6-bisphosphate
F6P: Fructose 6-phosphate
G6P: Glucose 6-phosphate
GAP: Glyeraldehyde 3-phosphate
GC: Gas chromatography
Gluc: Glucose
HPLC: High-performance liquid chromatography
IPS: Internal polysaccharides
Nqr: NADH:quinone oxidoreductase
OD_600_: Optical density at 600 nm
OD_max_: Maximal optical density
OxAc: Oxaloacetate
oxPPP: Oxidative branch of the pentose phosphate pathway
PEP: Phosphoenolpyruvate
PPP: Pentose phosphate pathway
Pro: Propionate
PYHVG: Peptone yeast hemin vitamin and glucose medium
R5P: Ribose 5-phosphate
RCB: Resting cells buffer
Rnf: Ferredoxin:NAD oxidoreductase
Ru5P: Ribulose 5-phosphate
S7P: Seduheptulose 7-phosphate
TCA: Tricarboxylic acid cycle
TRBA: Technical rule for biological agents
Wgs: Whole genome shotgun sequencing
Xu5P: Xylose 5-phosphate
