# Supplementary material for: Propionate production by Bacteroidia gut bacteria and its dependence on substrate concentrations differs among species
Source: Biotechnol Biofuels Bioprod. 2024 Jul 10;17:95. doi: 10.1186/s13068-024-02539-9 (PMC11238397; doi:10.1186/s13068-024-02539-9)
Supplement: Supplementary file 1 — Additional file 1: Table S1. Selection of Bacteroidia that produce propionate and respective plant polymers that can be utilized. Acids are listed in decreasing order according to relative amounts produced. Strains used in this study are written in bold. Abbreviations: acetate (A), propionate (P), succinate (S); lactate (L); malate (M), butyrate (B); isobutyrate (IB); isovalerate (IV), fumarate (Fu), not determined (Nd). [file 13068_2024_2539_MOESM1_ESM.pdf]

Table S1: Selection of *Bacteroidia* that produce propionate and respective plant polymers that can be utilized.

Organism	Strain	Isolated from	Acids produced	Polymeric substrates	Risc Group**	Reference
Bacteroides						
B. cellulosilyticus	**CRE21**	**Human feces**	**A, P, S, F, L**	**CWS, cellulose (Avicel p101, Sigmacell 101), starch, pectin**	**1**	**[1]**
B. fragilis	EN-2	Appendix abscess	S, A, P, L	*Starch	2	[2, 3]
B. graminisolvens	**XDT-1**	**Methanogenic reactor**	**A, P, S, L, B**	**Xylan, starch, pectin**	**1**	**[4]**
B. intestinalis	**341**	**Human feces**	**Fu, A, P**	**-**	**1**	**[5, 6];**
B. luti	**UasXn-3**	**Methanogenic sludge**	**A, S, P, F**	**Cellulose, xylan, starch**	**1**	**[5]**
B. ovatus	ATCC 8483	Human feces	A, P, S	Starch, pectin, xylan, arabinogalactan	2	[7, 8]
B. propionicifaciens	**SV434**	**Methanogenic reactor**	**A, P, S**	**Starch**	**1**	**[9]**
B. propionicigenes	NSJ-90	Human feces	A, P, IV	Starch, Xylan	Nd.	[10]
B. reticulotermitis	Rs-03	Termite gut	S, A, P	Xylan, Starch, CMC, pectin	1	[11]
B. thetaiotaomicron	E50		A, P, S	*Starch, pectin, polygalacturonate, larch arabinoglactan	2	[3, 12, 13]
B. xylanisolvens	**XB1A**	**Human feces**	**A, P, S**	**Xylan**	**1**	**[14]**
Butyricimonas						
B. faecalis	H184	Human feces	P, A, IB, B, IV, V	-	Nd.	[15]
Coprobacter						
C. fastidiosus	NSB1	Infant feces	A, P, S	Nd.	1	[16]
Dysgonomonas						
D. alginatilytica	HUA-2	Alginat degrading consortium	L, S, A, P	Alginate	1	[17]
D. capnocytophagoides	CCUG 17996	Infected human gallbladder	P, L, S	Nd.	2	[18]
D. mossii	CCUG 43457	Human clinical sample	L, A, P	Nd.	1	[19]

Fermentimonas						
F. caenicola	ING2-E5B	Mesophilic biogas reactor	A, P	Soluble starch	1	[20]
Lentimicrobium						
L. saccharophilum	TBC1	Methanogenic sludge	A, M, P, F	starch	1	[21]
Odoribacter						
O. splanchnicus	1651/6	Human feces	A, P, IB, B	Nd.	2	[22, 23]
Paludibacter						
P. propionicigenes	WB4	Anoxic rice-field soil	P, A, S	Soluble starch	1	[24]
P. jianxiensis	NM7	rice paddy field	A, P	Cellobiose, pectin, starch	1	[25]
Parabacteroides						
P. acidifaciens	426-9	Human feces	A, P, IB	Nd.	Nd.	[26]
P. chartae	**NS31-3**	**Paper mill wastewater**	**L, P, F, A**	**Nd.**	**1**	**[27]**
P. johnsonii	**M-165**	**Human feces**	**S, A, P**	**Nd.**		**[28]**
Petrimonas						
P. mucosa	ING2-E5A	Mesophilic biogas reactor	A, P	Soluble starch	1	[20]
Phocaeicola						
P. paurosaccharolyticus	**WK042**	**Methanogenic reactor**	**A, P, S**	**Glycogen, starch, pectin**	**1**	**[29, 30]**
P. vulgatus	**ATCC 8482**	**Human feces**	**A, S, F, L, P**	***Starch, larch arabinogalactan**	**1**	**[3, 30, 31]**
Proteiniphilum						
P. sachcharofermentans	M3/6	Mesophilic biogas reactor	A, P	Soluble starch	1	[20]
Xylanibacter						
X. ruminicola	23	rumen	P, A, S		2	[32, 33]
X. oryzae	KB3	anoxic rice-field soil	A, P, S, M	Xylan, pectin, CMC	1	[34, 35]

Acids are listed in decreasing order according to relative amounts produced. Strains used in this study are written in bold. Abbreviations: acetate (A), propionate (P), succinate (S); lactate (L); malate (M), butyrate (B); isobutyrate (IB); isovalerate (IV), Fumarate (Fu), not determined (Nd.)

*Utilization has been confirmed for several strains. **According to German technical rule for biological agents 2015.

References

1. Robert C, Chassard C, Lawson PA, Bernalier-Donadille A. *Bacteroides cellulosilyticus* sp. nov., a cellulolytic bacterium from the human gut microbial community. Int J Syst Evol Microbiol. 2007;57:1516–20.
2. Chen M, Wolin MJ. Influence of heme and vitamin B_{12} on growth and fermentations of *Bacteroides* species. J Bacteriol. 1981;145:466–71.
3. Salyers AA, Vercellotti JR, West SE, Wilkins TD. Fermentation of mucin and plant polysaccharides by strains of *Bacteroides* from the human colon. Appl Environ Microbiol. 1977;33:319–22.
4. Nishiyama T, Ueki A, Kaku N, Watanabe K, Ueki K. *Bacteroides graminisolvens* sp. nov., a xylanolytic anaerobe isolated from a methanogenic reactor treating cattle waste. Int J Syst Evol Microbiol. 2009;59:1901–7.
5. Hatamoto M, Kaneshige M, Nakamura A, Yamaguchi T. *Bacteroides luti* sp. nov., an anaerobic, cellulolytic and xylanolytic bacterium isolated from methanogenic sludge. Int J Syst Evol Microbiol. 2014;64:1770–4.
6. Bakir MA, Kitahara M, Sakamoto M, Matsumoto M, Benno Y. *Bacteroides intestinalis* sp. nov., isolated from human faeces. Int J Syst Evol Microbiol. 2006;56:151–4.
7. Macfarlane GT, Hay S, Macfarlane S, Gibson GR. Effect of different carbohydrates on growth, polysaccharidase and glycosidase production by *Bacteroides ovatus*, in batch and continuous culture. J Appl Bacteriol. 1990;68:179–87.
8. Horvath TD, Ihekweazu FD, Haidacher SJ, Ruan W, Engevik KA, Fultz R, *et al.* *Bacteroides ovatus* colonization influences the abundance of intestinal short chain fatty acids and neurotransmitters. iScience. 2022;25:104158.
9. Ueki A, Abe K, Kaku N, Watanabe K, Ueki K. *Bacteroides propionicifaciens* sp. nov., isolated from rice-straw residue in a methanogenic reactor treating waste from cattle farms. Int J Syst Evol Microbiol. 2008;58:346–52.
10. Sun X-W, Abdugheni R, Huang H-J, Wang Y-J, Jiang M-Z, Liu C, *et al. Bacteroides propionicigenes sp.* nov., isolated from human faeces. Int J Syst Evol Microbiol 2022.
11. Sakamoto M, Ohkuma M. *Bacteroides reticulotermitis sp.* nov., isolated from the gut of a subterranean termite (*Reticulitermes speratus*). Int J Syst Evol Microbiol. 2013;63:691–5.
12. Kotarski SF, Salyers AA. Effect of long generation times on growth of *Bacteroides thetaiotaomicron* in carbohydrate-induced continuous culture. J Bacteriol. 1981;146:853–60.
13. Adamberg S, Tomson K, Vija H, Puurand M, Kabanova N, Visnapuu T, *et al.* Degradation of fructans and production of propionic acid by *Bacteroides thetaiotaomicron* are enhanced by the shortage of amino acids. Front Nutr. 2014;1:21.
14. Chassard C, Delmas E, Lawson PA, Bernalier-Donadille A. *Bacteroides xylanisolvens* sp. nov., a xylan-degrading bacterium isolated from human faeces. Int J Syst Evol Microbiol. 2008;58:1008–13.
15. Le Roy T, van der Smissen P, Paquot A, Delzenne N, Muccioli GG, Collet J-F, Cani PD. *Butyricimonas faecalis sp.* nov., isolated from human faeces and emended description of the genus *Butyricimonas*. Int J Syst Evol Microbiol. 2019;69:833–8.
16. Shkoporov AN, Khokhlova EV, Chaplin AV, Kafarskaia LI, Nikolin AA, Polyakov VY, *et al. Coprobacter fastidiosus* gen. nov., sp. nov., a novel member of the family

Porphyromonadaceae isolated from infant faeces. Int J Syst Evol Microbiol. 2013;63:4181–8.

17. Kita A, Miura T, Okamura Y, Aki T, Matsumura Y, Tajima T, *et al. Dysgonomonas alginatilytica* sp. nov., an alginate-degrading bacterium isolated from a microbial consortium. Int J Syst Evol Microbiol. 2015;65:3570–5.
18. Hofstad T, Olsen I, Eribe ER, Falsen E, Collins MD, Lawson PA. *Dysgonomonas* gen. nov. to accommodate *Dysgonomonas gadei* sp. nov., an organism isolated from a human gall bladder, and *Dysgonomonas capnocytophagoides* (formerly CDC group DF-3). Int J Syst Evol Microbiol. 2000;50 Pt 6:2189–95.
19. Lawson PA, Falsen E, Inganäs E, Weyant RS, Collins MD. *Dysgonomonas mossii* sp. nov., from human sources. Syst Appl Microbiol. 2002;25:194–7.
20. Hahnke S, Langer T, Koeck DE, Klocke M. Description of *Proteiniphilum saccharofermentans* sp. nov., *Petrimonas mucosa* sp. nov. and *Fermentimonas caenicola* gen. nov., sp. nov., isolated from mesophilic laboratory-scale biogas reactors, and emended description of the genus *Proteiniphilum*. Int J Syst Evol Microbiol. 2016;66:1466–75.
21. Sun L, Toyonaga M, Ohashi A, Tourlousse DM, Matsuura N, Meng X-Y, *et al. Lentimicrobium saccharophilum* gen. nov., sp. nov., a strictly anaerobic bacterium representing a new family in the phylum Bacteroidetes, and proposal of Lentimicrobiaceae fam. nov. Int J Syst Evol Microbiol. 2016;66:2635–42.
22. Werner H, Rintelen G, Kunstek-Santos H. Eine neue buttersäurebildende *Bacteroides-Art*: *B. splanchnicus* n. sp [A new butyric acid-producing *bacteroides* species: *B. splanchnicus* n. sp.]. Zentralbl Bakteriol Orig A. 1975;231:133–44.
23. Hardham JM, King KW, Dreier K, Wong J, Strietzel C, Eversole RR, *et al.* Transfer of *Bacteroides splanchnicus* to *Odoribacter* gen. nov. as *Odoribacter splanchnicus* comb. nov., and description of *Odoribacter denticanis* sp. nov., isolated from the crevicular spaces of canine periodontitis patients. Int J Syst Evol Microbiol. 2008;58:103–9.
24. Ueki A, Akasaka H, Suzuki D, Ueki K. *Paludibacter propionicigenes* gen. nov., sp. nov., a novel strictly anaerobic, Gram-negative, propionate-producing bacterium isolated from plant residue in irrigated rice-field soil in Japan. Int J Syst Evol Microbiol. 2006;56:39–44.
25. Qiu Y-L, Kuang X-Z, Shi X-S, Yuan X-Z, Guo R-B. *Paludibacter jiangxiensis* sp. nov., a strictly anaerobic, propionate-producing bacterium isolated from rice paddy field. Arch. Microbiol. 2014;196:149–55.
26. Wang Y-J, Xu X-J, Zhou N, Sun Y, Liu C, Liu S-J, You X. *Parabacteroides acidifaciens* sp. nov., isolated from human faeces. Int J Syst Evol Microbiol. 2019;69:761–6.
27. Tan H-Q, Li T-T, Zhu C, Zhang X-Q, Wu M, Zhu X-F. *Parabacteroides chartae* sp. nov., an obligately anaerobic species from wastewater of a paper mill. Int J Syst Evol Microbiol. 2012;62:2613–7.
28. Sakamoto M, Kitahara M, Benno Y. *Parabacteroides johnsonii* sp. nov., isolated from human faeces. Int J Syst Evol Microbiol. 2007;57:293–6.
29. Ueki A, Abe K, Ohtaki Y, Kaku N, Watanabe K, Ueki K. *Bacteroides paurosaccharolyticus* sp. nov., isolated from a methanogenic reactor treating waste from cattle farms. Int J Syst Evol Microbiol. 2011;61:448–53.
30. García-López M, Meier-Kolthoff JP, Tindall BJ, Gronow S, Woyke T, Kyrpides NC, *et al.* Analysis of 1,000 type-strain genomes improves taxonomic classification of *Bacteroidetes*. Front Microbiol. 2019;10:2083.

31. Lück R, Deppenmeier U. Genetic tools for the redirection of the central carbon flow towards the production of lactate in the human gut bacterium *Phocaeicola (Bacteroides) vulgatus*. Appl Microbiol Biotechnol. 2022;106:1211–25.
32. Strobel HJ. Vitamin B12-dependent propionate production by the ruminal bacterium *Prevotella ruminicola* 23. Appl Environ Microbiol. 1992;58:2331–3.
33. Hitch TCA, Bisdorf K, Afrizal A, Riedel T, Overmann J, Strowig T, Clavel T. A taxonomic note on the genus *Prevotella*: Description of four novel genera and emended description of the genera *Hallella* and *Xylanibacter*. Syst Appl Microbiol. 2022;45:126354.
34. Ueki A, Akasaka H, Suzuki D, Hattori S, Ueki K. *Xylanibacter oryzae* gen. nov., sp. nov., a novel strictly anaerobic, Gram-negative, xylanolytic bacterium isolated from rice-plant residue in flooded rice-field soil in Japan. Int J Syst Evol Microbiol. 2006;56:2215–21.
35. Sakamoto M, Ohkuma M. Reclassification of *Xylanibacter oryzae* Ueki *et al*. 2006 as *Prevotella oryzae* comb. nov., with an emended description of the genus *Prevotella*. Int J Syst Evol Microbiol. 2012;62:2637–42.
