# Supplementary material for: Propionate production by Bacteroidia gut bacteria and its dependence on substrate concentrations differs among species
Source: Biotechnol Biofuels Bioprod. 2024 Jul 10;17:95. doi: 10.1186/s13068-024-02539-9 (PMC11238397; doi:10.1186/s13068-024-02539-9)
Supplement: Supplementary file 2 — Additional file 2: Table S2. Growth parameters of ten strains of the class Bacteroidia. Growth was determined by measurement of OD600 every 15 minutes for 24 h (45 h in case of B. propionicifaciens) in defined minimal medium with 15 mM glucose (DMMG) without yeast extract (-) or with 0.5 g/l yeast extract (+). The experiment was carried out in a plate reader set to 30°C or 37°C, respectively, placed in an anoxic chamber (Coy laboratory products, Grass Lake, USA). Mean values and standard deviation of three biological replicates are shown. ODmax was the highest OD600 value measured. The specific growth rates were determined from 5 consecutive timepoints by a regression analysis. Table S3. Product formation of two strains of the genus Parabacteroides and Phocaeicola. Cells were grown for 24 h (48 h for B. propionicifaciens) in defined minimal medium with 15 mM glucose (DMMG), either with (+) or without (-) 0.5 g/l yeast extract. Samples for product formation were retrieved at the end and analysed via HPLC. Average values and standard deviation of three biological replicates are shown. Fig. S1. Growth and remaining glucose concentration of 10 Bacteroidia cultures in DMMG with 15 mM glucose. (A) OD600 was measured every 15 minutes with a plate reader for up to 45 h (every second measurement shown). One representative curve of three is shown. (B) Glucose concentration after 24 h of cultivation. A second measurement was performed with B. propionicifaciens cultures after 45 h. Average and standard deviation of three biological replicates are shown. Fig. S2. Gas content in the headspace of B. graminisolvens, B. propionicifaciens, S. copri and B. cellulosilyticus. Cells were grown in defined minimal medium with either 15 mM (turquoise) or 30 mM of glucose (black) until stationary growth phase. Samples of the control gases and the headspace of the medium control are depicted in blue. [file 13068_2024_2539_MOESM2_ESM.pdf]

Additional File 2

Table S2: Growth parameters of ten strains of the class *Bacteroidia*. Growth was determined by measurement of OD_{600} every 15 minutes for 24 h (45 h in case of *B. propionicifaciens*) in defined minimal medium with 15 mM glucose (DMMG) without yeast extract (-) or with 0.5 g/l yeast extract (+). The experiment was carried out in a plate reader set to 30 or 37 °C respectively, placed in an anoxic chamber (Coy laboratory products, Grass Lake, USA). Mean values and standard deviation of three biological replicates are shown. OD_{max} was the highest OD_{600} value measured. The specific growth rates were determined from 5 consecutive timepoints by a regression analysis.

Strain	Yeast extract	OD_{max}	Growth rate [h^{-1}]
Bacteroides			
B. cellulosilyticus CRE21	-	**0.80** ± 0.09	**0.44** ± 0.01
	+	**0.82** ± 0.04	**0.5** ± 0.00
B. graminisolvens XDT-1	-	**0.73** ± 0.04	**0.27** ± 0.02
	+	**0.73** ± 0.03	**0.28** ± 0.03
B. intestinalis 341	-	**0.88** ± 0.02	**0.44** ± 0.01
	+	**0.88** ± 0.08	**0.48** ± 0.01
B. luti UasXn-3	-	**0.58** ± 0.01	**0.16** ± 0.02
	+	**0.59** ± 0.04	**0.16** ± 0.01
B. xylanisolvens XB1A	-	**0.78** ± 0.00	**0.50** ± 0.08
	+	**0.81** ± 0.00	**0.61** ± 0.01
B. propionicifaciens SV434	-	**0.68** ± 0.03	**0.09** ± 0.00
	+	**0.76** ± 0.02	**0.14** ± 0.00
Parabacteroides			
P. chartae NS31-3	-	**0.86** ± 0.02	**0.36** ± 0.05
	+	**0.87** ± 0.03	**0.35** ± 0.00
P. johnsonii M-165	-	**0.65** ± 0.00	**0.23** ± 0.02
	+	**0.73** ± 0.00	**0.32** ± 0.00
Phocaeicola			
P. paurosaccharolyticus WK042	-	**0.90** ± 0.01	**0.16** ± 0.01
	+	**0.92** ± 0.02	**0.17** ± 0.00
P. vulgatus ATCC 8482	-	**0.88** ± 0.03	**0.33** ± 0.03
	+	**0.92** ± 0.02	**0.39** ± 0.01

Table S3: Product formation of two strains of the genus *Parabacteroides* and *Phocaeicola*. Cells were grown for 24 h in defined minimal medium with 15 mM glucose (DMMG), either with (+) or without (-) 0.5 g/l yeast extract. Samples for product formation were retrieved at the end and analysed via HPLC. Average values and standard deviation of three biological replicates are shown.

		Products [mM]			
**Yeast extract [+/-]**		***P. chartae*** NS31-3	***P. johnsonii*** M-165	***P. paurosaccharolyticus*** WK042	***P. vulgatus*** ATCC 8482
	**Propionate**				
-		**6.4** ± 0.31	**5.0** ± 0.62	**4.1** ± 0.35	**3.4** ± 0.40
+		**4.9** ± 1.10	**6.4** ± 0.24	**5.0** ± 0.21	**4.0** ± 0.04
	**Acetate**				
-		**7.7** ± 0.08	**6.7** ± 0.92	**5.6** ± 0.47	**9.9** ± 0.51
+		**7.9** ± 0.14	**7.5** ± 0.36	**6.0** ± 0.20	**10.2** ± 0.37
	**Succinate**				
-		**3.5** ± 0.22	**3.6** ± 0.60	**3.7** ± 0.30	**3.7** ± 0.35
+		**3.7** ± 0.57	**3.0** ± 0.17	**4.0** ± 0.35	**4.2** ± 0.35
	**Lactate**				
-		**0.0** ± 0.00	**0.0** ± 0.00	**0.5** ± 0.09	**0.0** ± 0.00
+		**0.2** ± 0.21	**0.0** ± 0.00	**0.6** ± 0.05	**0.0** ± 0.00
	**Formate**				
-		**4.0** ± 0.46	**0.3** ± 0.21	**0.9** ± 0.46	**1.1** ± 0.14
+		**4.4** ± 0.20	**0.4** ± 0.02	**0.9** ± 0.40	**1.1** ± 0.07
	**Yield [g_{pro}/g_{gluc}]**				
-		**0.16** ± 0.02	**0.15** ± 0.01	**0.11** ± 0.04	**0.08** ± 0.01
+		**0.13** ± 0.03	**0.20** ± 0.00	**0.14** ± 0.00	**0.10** ± 0.00
	**Molar ratio [mol_{pro}/mol_{suc})**				
-		**1.9**	**1.4**	**1.1**	**0.9**
+		**1.4**	**2.2**	**1.3**	**0.9**
	**Molar ratio [(mol_{pro} +mol_{suc}) /mol_{ac}]**				
-		**1.3**	**1.3**	**1.4**	**0.7**
+		**1.1**	**1.3**	**1.5**	**0.8**

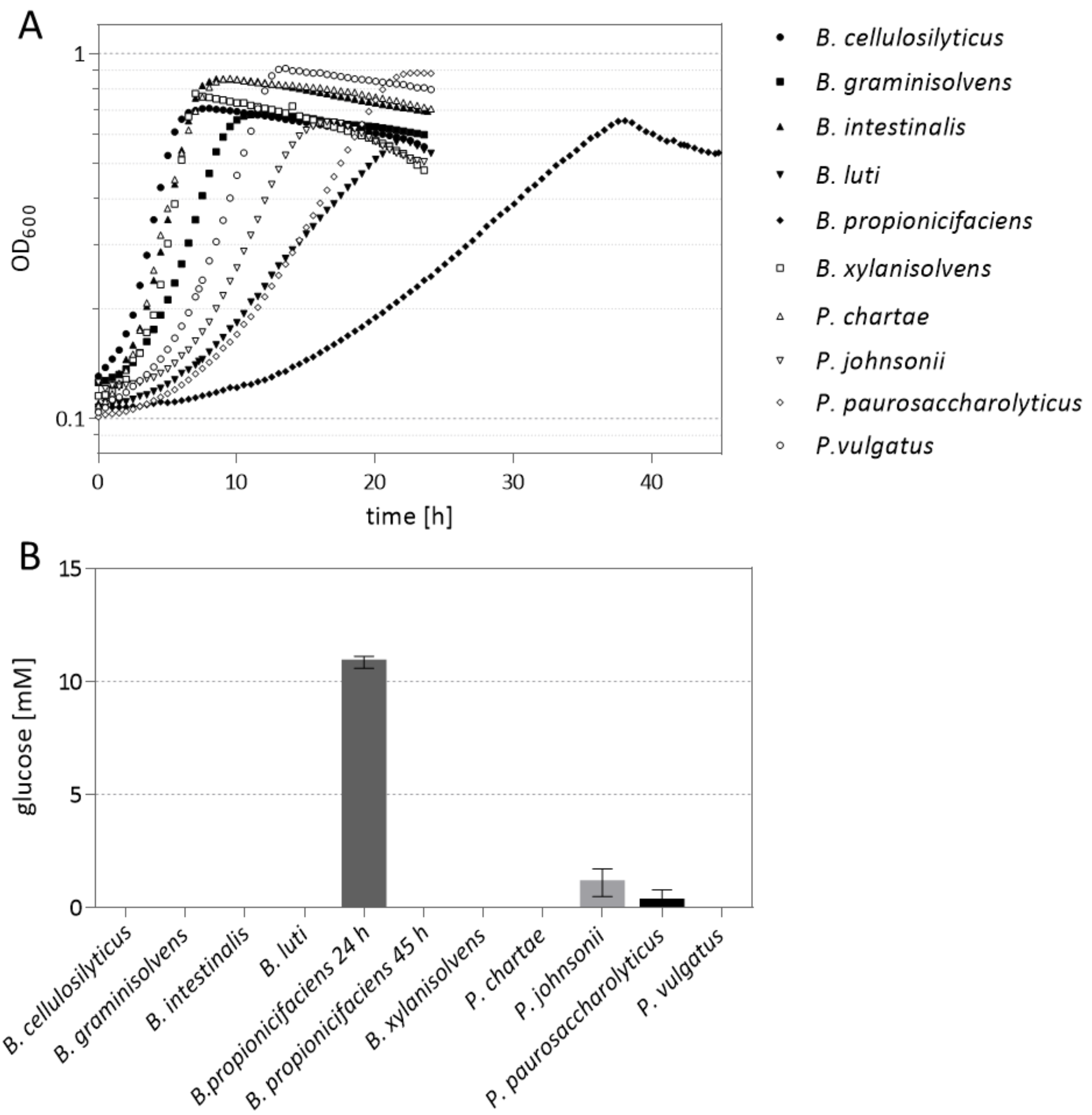

Fig. S1: Growth and remaining glucose concentration of 10 *Bacteroidia* cultures in DMMG with 15 mM glucose. (A) OD_{600} was measured every 15 minutes with a plate reader for up to 45 h (every second measurement shown). One representative curve of three is shown. (B) Glucose concentration after 24 h of cultivation. A second measurement was performed with *B. propionicifaciens* cultures after 45 h. Mean values and standard deviation of three biological replicates are shown.

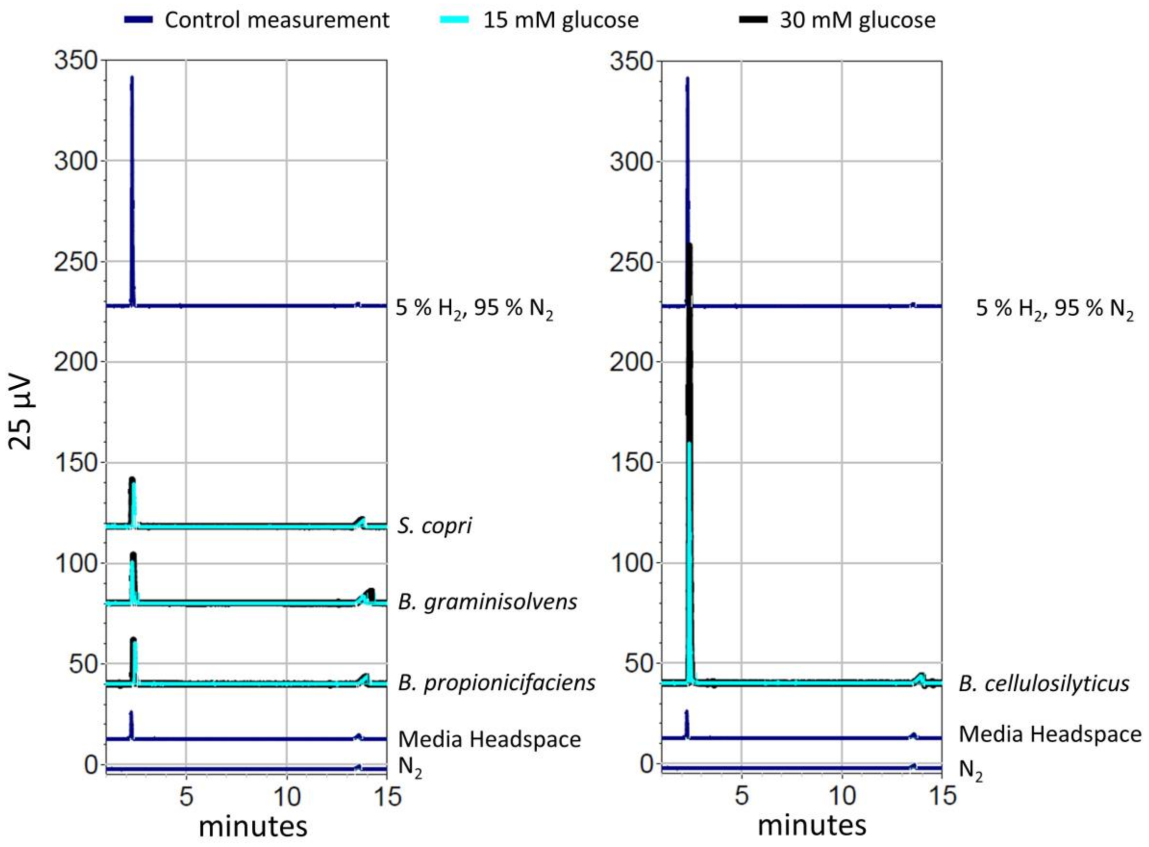

Fig. S2 Gas content in the headspace of *B. graminisolvens*, *B. propionicifaciens*, *S. copri* and *B. cellulosilyticus*. Cells were grown in defined minimal medium with either 15 mM (turquoise) or 30 mM of glucose (black) until stationary growth phase. Samples of the control gases and the headspace of the medium control are depicted in blue.
