# Supplementary material for: Propionate production by Bacteroidia gut bacteria and its dependence on substrate concentrations differs among species
Source: Biotechnol Biofuels Bioprod. 2024 Jul 10;17:95. doi: 10.1186/s13068-024-02539-9 (PMC11238397; doi:10.1186/s13068-024-02539-9)
Supplement: Supplementary file 4 — Additional file 4: Table S6. Components of SL-6 trace element solution per litre. Table S7. Components of 5 x Wolin solution per litre. [file 13068_2024_2539_MOESM4_ESM.pdf]

Tab. S5: Components of SL-6 trace element solution per litre

**Substance**	**amount**
$ZnSO_4$ x $7H_2O$	0,1 g
$MnCl_2$ x $4H_2O$	30 mg
H_3BO_3	0,3 g
$CoCl_2$ x $6H_2O$	0,2 g
$CuCl_2$ x $2H_2O$	10 mg
$NiCl_2$ x $6H_2O$	20 mg
Na_2MoO_4 x $2H_2O$	30 mg
H_2O_{dest}	ad 1000 ml

Tab. S6: Components of 5 x Wolin solution per litre

**Substance**	**amount**
Biotin	10 mg
Folic acid	10 mg
Pyridoxine x HCl	50 mg
Thiamine x HCl	25 mg
Na-Riboflavin	25 mg
Nicotinic acid	25 mg
Ca-Pantothenate	25 mg
Vitamine B12	21 mg
p-Aminobenzoic acid	25 mg
α-Lipoic acid	25 mg
H_2O_{dest}	ad 1000 ml

pH was adjusted to 7.5 with NaOH.
